# Negative Impact of p21‐Activated Kinase 4‐Mediated AMP‐Activated Protein Kinase Inhibition on Sarcopenia in Mice and Humans

**DOI:** 10.1002/mco2.70508

**Published:** 2025-11-29

**Authors:** Jiacheng Du, Hwang Chan Yu, Young Jae Moon, Sun‐Jung Yoon, Seung‐Yong Seo, Byung‐Hyun Park, Eun Ju Bae

**Affiliations:** ^1^ Department of Biochemistry and Molecular Biology Jeonbuk National University Medical School Jeonju Republic of Korea; ^2^ Graduate School of Medical Science and Engineering, Korea Advanced Institute of Science and Technology Daejeon Republic of Korea; ^3^ Department of Orthopedic Surgery Jeonbuk National University Hospital Jeonju Republic of Korea; ^4^ College of Pharmacy Gachon University Incheon Republic of Korea; ^5^ School of Pharmacy and Institute of New Drug Development Jeonbuk National University Jeonju Republic of Korea

**Keywords:** AMPK, mitochondria, muscle atrophy, p21‐activated kinase 4, proteolysis‐targeting chimera

## Abstract

We recently identified that AMP‐activated protein kinase (AMPK) α2 phosphorylation at S491 is mediated by p21‐activated kinase 4 (PAK4), leading to muscular and systemic insulin resistance. This study examined how muscle PAK4 deletion affects atrophy in male mice and its link to human sarcopenia. Dexamethasone treatment increased the mRNA and protein levels of PAK4, which was partially the result of glucocorticoid response elements activation in the promoter of the *Pak4* gene. Muscle‐specific *Pak4* knockout mice were protected from both dexamethasone‐ and denervation‐induced muscle atrophy. Likewise, treatment with a proteolysis‐targeting chimera (PROTAC) targeting PAK4 also mitigated muscle atrophy. PAK4 inhibition alleviated mitochondrial dysfunction and enhanced the expression of biogenesis‐related genes via AMPK activation with reduced AMPKα2‐S491 phosphorylation. Notably, muscle overexpression of phospho‐deficient AMPKα2^S491A^ mutant preserved mass in dexamethasone‐treated mice, whereas constitutively phosphorylated AMPKα2^S491D^ mutant abolished PAK4 PROTAC's antiatrophy effect. In humans, sarcopenic muscle exhibited higher levels of PAK4 protein and AMPKα2‐S491 phosphorylation compared with non‐sarcopenia controls, with an inverse correlation to sarcopenic index and grip strength. These findings reveal a novel AMPK phosphorylation‐dependent mechanism by which PAK4 regulates mitochondrial function and muscle mass, offering new therapeutic avenues for combating muscle atrophy in chronic disease and aging.

**Clinical trial registration**: Not applicable.

## Graphical abstract

- PAK4 is overexpressed in muscle atrophy and negatively correlates with muscle mass and function in humans.
- Inhibiting PAK4 activates AMPK/PGC‐1α‐driven mitochondrial biogenesis by blocking AMPKα2‐S491 phosphorylation while promoting muscle growth signals.
- PROTAC targeting PAK4 is a potential treatment for sarcopenia.

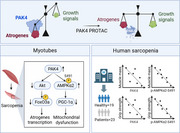

## 1 Introduction

Skeletal muscle atrophy, commonly referred to as sarcopenia, is marked by a reduction in myofiber size due to the loss of structural proteins, organelles, and cytoplasmic content. It is primarily linked to aging, physical inactivity, and adverse effects of certain medications, including glucocorticoids, statins, and specific anti‐cancer drugs [1]. With shifting demographics and rising life expectancy, the prevalence of sarcopenia continues to increase in modern societies. A comprehensive meta‐analysis of 263 studies, encompassing over 690,000 participants, estimated that sarcopenia affects 10% to 27% of adults aged 60 and older [2]. This condition imposes a significant societal and economic burden while diminishing the quality of life in older adults. Sarcopenic individuals face a heightened risk of frailty, falls, morbidity, and mortality. Currently, there are no Food and Drug Administration‐approved treatments for sarcopenia, with only high‐protein diets and resistance training demonstrating clinical efficacy [3]. Therefore, further research into its pathophysiology and the development of novel therapies remain essential to mitigating sarcopenia and its associated health risks.

The etiology and pathophysiology of sarcopenia are complex and multifactorial. Well‐established contributors include an imbalance between protein synthesis and degradation [4], mitochondrial dysfunction [5], excess intramuscular fat accumulation [6], and shifts in muscle fiber type [7]. However, alterations in these mechanisms vary across studies, and their exact contribution to muscle function loss remains unclear. Nonetheless, recent research underscores the pivotal role of mitochondrial dysfunction in the development of sarcopenia. Several mitochondria‐associated mechanisms contribute to this process, including excessive reactive oxygen species production, disruptions in mitochondrial dynamics (fission and fusion), reduced mitochondrial biogenesis, and impaired regulation of autophagy and mitophagy [1].

AMP‐activated protein kinase (AMPK), a heterotrimeric energy sensor, is pivotal in skeletal muscle metabolism, and its activity is primarily regulated by phosphorylation at T172 on the catalytic α1/α2 subunits. AMPK, in turn, phosphorylates and activates peroxisome proliferator‐activated receptor gamma (PPARγ) coactivator‐1 alpha (PGC‐1α) [8], a critical regulator of mitochondrial biogenesis and homeostasis that protects against muscle atrophy. Consistently, pharmacological AMPK activation with 5‐aminoimidazole‐4‐carboxamide ribonucleoside (AICAR) enhances PGC‐1α activity and prevents muscle wasting [8, 9]. AMPK can also stimulate PGC‐1α via Sirtuin 1 (Sirt1)—a NAD⁺‐dependent protein deacetylase‐mediated deacetylation, recapitulating exercise‐induced benefits [10, 11]. Conversely, AMPK activation may suppress anabolic signaling by inhibiting mammalian target of rapamycin (mTOR) and upregulating muscle‐specific E3 ubiquitin ligases [12]. While the AMPK–PGC‐1α signaling may help counteract skeletal muscle atrophy, AMPK–mTOR inhibition or AMPK–ubiquitin proteasomal system may accelerate sarcopenia. This dual role of AMPK in sarcopenic muscle wasting presents an apparent paradox that remains to be resolved. Additionally, substantial evidence suggests that AMPK contributes to reducing intramuscular fat contents [13, 14] and promotes the shift of muscle fiber types from fast, glycolytic to slow, oxidative myofibers [15]. This transition could alter myofiber composition, potentially compromising muscle mass and function.

p21‐activated kinase 4 (PAK4), an effector molecule of Rho GTPases such as RhoA, Cdc42, and Rac1, is well recognized as an oncoprotein that orchestrates cytoskeletal dynamics, tumor progression, and metastasis [16]. Beyond its established role in cancer, our previous studies have revealed several novel physiological functions of PAK4. Specifically, PAK4 has been shown to regulate oxidative stress during hepatic and renal ischemia–reperfusion injury [17, 18], control hepatic ketogenesis during fasting [19], and modulate triglyceride lipolysis and adipocyte differentiation in adipose tissue [20, 21]. Furthermore, we identified PAK4 as a negative regulator of AMPK activity in skeletal muscle. Mechanistically, PAK4 inhibits AMPK by phosphorylating AMPKα1‐S485 and AMPKα2‐S491, while also suppressing the Akt signaling pathway [22]. As a consequence, GLUT4 expression and translocation are reduced, leading to impaired glucose uptake in muscle. In addition, PAK4 phosphorylates PPARγ‐S273, inducing phosphatase and tensin homolog (PTEN) transcription and thus inhibiting PI3K/Akt‐driven muscle regeneration [23], collectively contributing to diminished metabolic flexibility and impaired repair capacity. Consequently, genetic ablation or pharmacological inhibition of PAK4 enhances AMPK‐ and Akt‐mediated signaling, improves mitochondrial and glucose metabolism in muscle fibers, and promotes myogenesis under stress conditions.

Building on these observations, we hypothesized that therapeutic targeting of PAK4 could mitigate muscle atrophy by restoring AMPK activity and reinforcing muscle regenerative pathways. To test this hypothesis, we generated muscle‐specific *Pak4* knockout (KO) mice and systematically evaluated their resistance to glucocorticoid– or sciatic nerve denervation–induced atrophy. Furthermore, we evaluated the efficacy of the PAK4‐targeted proteolysis‐targeting chimera (PROTAC) SJ‐05, which induces selective protein degradation [17], and examined PAK4 expression as well as its regulation of AMPK phosphorylation in human muscle tissues with sarcopenia. Together, these approaches aim to establish PAK4 not only as a molecular link between metabolism and muscle homeostasis but also as a promising therapeutic target for the prevention of sarcopenia and related muscle‐wasting disorders.

## 2 Results

### 2.1 Muscle Atrophy Is Associated With the Increased PAK4 Protein Levels

**FIGURE 1:**
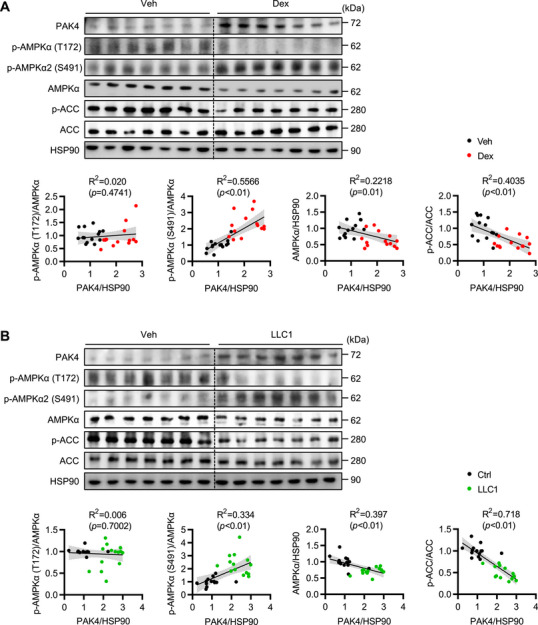
Increased p21‐activated kinase 4 (PAK4) expression in sarcopenic mouse models. Protein levels of PAK4 and AMPKα signaling in gastrocnemius muscles: (A) 10 days after intraperitoneal injection of dexamethasone (Dex) at 25 mg/kg and (B) 21 days after subcutaneous implantation of LLC1 cells in C57BL/6J mice. Gastrocnemius muscles were removed for the analysis. Correlation between the PAK4 and AMPKα signaling proteins (*n* = 28).

To determine whether the PAK4 protein level is altered under sarcopenic conditions, we examined it in different mouse models of muscle atrophy. In male C57BL/6J mice, dexamethasone treatment increased PAK4 protein levels in gastrocnemius muscles compared with the control group, while other PAK family members remained unchanged (Figure 1A, S1A). We further compared two key phosphorylation sites of AMPKα: T172, a positive regulatory modification, and S485/491 (AMPKα1/α2), a negative regulatory modification. Dexamethasone treatment increased AMPKα2 phosphorylation at S491, a known PAK4 substrate [22], whereas phosphorylation at T172 and AMPK activity, assessed by p‐ACC levels, were reduced (Figure 1A). PAK4 levels positively correlated with AMPKα2‐S491 phosphorylation but negatively with total AMPKα protein. Selective elevation of PAK4 protein, with no changes in other PAK members, and AMPKα2 phosphorylation at S491 were consistently observed in various muscle atrophy models, including those induced by cancer cachexia, hindlimb suspension, and aging (Figure 1B; Figure S1B–D). This was accompanied by a pronounced induction of MuRF1 and Atrogin‐1, key regulators of muscle atrophy [24], along with the suppression of the myogenic transcription factor myogenin (Figure S2A–D).

We then investigated whether dexamethasone directly affected PAK4 expression. In C2C12 myotubes, dexamethasone treatment elevated PAK4 mRNA and protein levels in a concentration‐ and time‐dependent manner (Figure S3A–D). Since *Pak4* transcript levels were altered, we analyzed the promoter of mouse and human *Pak4* genes using the Evolutionary Conserved Regions (ECR) browser to test if a glucocorticoid response element (GRE) exists. GRE was newly recognized in the promoter regions of PAK4, with conserved binding motifs across human and murine species (Figure S3E). Genotype‐Tissue Expression (GTEx) database examination identified a positive correlation between human PAK4 expression and glucocorticoid receptor *NR3C1* expression (Figure S3F). Consistently, dexamethasone enhanced PAK4 promoter luciferase reporter activity (Figure S3G), but showed no stimulation in the putative GRE‐deletion mutant PAK4‐promoter reporter assay (Figure S3H).

We further explored the post‐transcriptional mechanism of PAK4 protein upregulation in muscle atrophy, as PAK4 was increased in all the different atrophy models. Since our previous study demonstrated that cAMP/protein kinase A (PKA) signaling and Sirt1 independently mediate PAK4 protein destabilization via ubiquitination and proteasomal pathways [19, 20], we assessed PKA activity and Sirt1 levels in gastrocnemius muscles. Our results showed that PKA activity (phosphorylation of PKA substrates) and Sirt1 protein levels were reduced across multiple muscle atrophy models (Figure S3I–L). These findings suggest that PAK4 protein levels are upregulated through both transcriptional and post‐transcriptional mechanisms in various catabolic conditions associated with muscle loss.

### 2.2 Skeletal Muscle‐Specific PAK4 Deficiency Mitigates Muscle Atrophy

**FIGURE 2:**
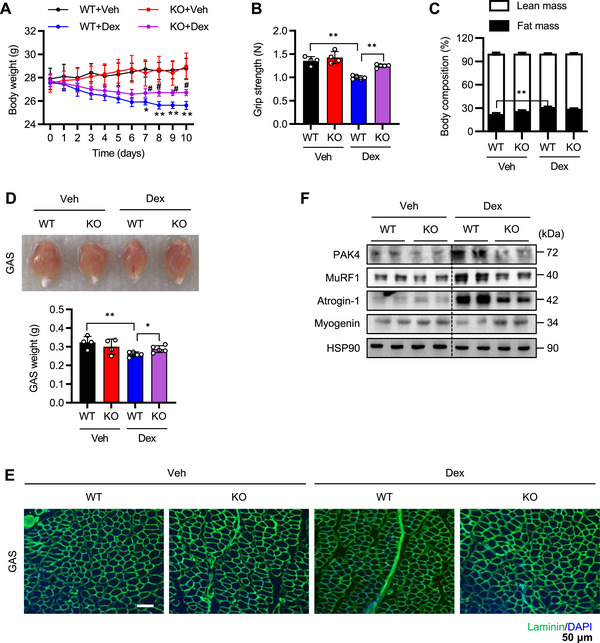
Mitigation of dexamethasone‐induced muscle atrophy by PAK4 deficiency. (A) Skeletal muscle‐specific *Pak4* KO mice and their wild‐type (WT) littermates were intraperitoneally administered dexamethasone (Dex, 25 mg/kg) daily for 10 days, and body weight was monitored over time. ^*^ *p *< 0.05 and ^**^ *p *< 0.01 vs. WT+Veh; ^#^, *p*<0.05 vs. WT+Dex. (B, C) At the end of the study, forelimb grip strength was evaluated at 15 min intervals, and lean body mass was determined using an NMR analyzer. (D) Representative images of gastrocnemius (GAS) muscle morphology and wet weight. (E) Immunofluorescence staining of laminin in muscle sections. Bar = 50 µm. (F) PAK4 and atrophy‐related proteins were analyzed by Western blotting. Values are mean ± SD (*n* = 4 for Veh, *n* = 5 for Dex). ^*^ *p *< 0.05 and ^**^ *p *< 0.01.

We next examined whether PAK4 directly influences skeletal muscle mass and function during atrophy by treating *Pak4* KO mice and their WT littermates with dexamethasone for 10 days. Dexamethasone‐treated mice exhibited a significant reduction in body weight, muscle strength (as measured by grip strength), and lean mass, along with an increase in fat mass compared with vehicle‐treated mice (Figure 2A–C). However, *Pak4* KO mice displayed an increased body weight despite similar food intake (Figure S4A) and were resistant to dexamethasone‐induced muscle atrophy. These findings were further supported by analyses of gross morphology, wet weight, myofiber diameter, and Western blotting and qPCR for MuRF1 and Atrogin‐1 in gastrocnemius muscle (Figure 2D–F; Figure S4B–D). Additionally, the wet weights of tibialis anterior (TA) and extensor digitorum longus (EDL) muscles were higher in *Pak4* KO mice compared with WT mice following dexamethasone treatment (Figure S4E,F), while the soleus (SOL) muscle exhibited a nonsignificant trend toward increased weight (Figure S4G).

Conversely, adenoviral overexpression of PAK4 in muscle, but not the kinase‐dead mutant PAK4^S474A^, was associated with reduced gastrocnemius mass, AMPK activity, and mitochondrial oxidative phosphorylation (OxPhos) proteins in response to dexamethasone treatment (Figure S5A–G).

**FIGURE 3:**
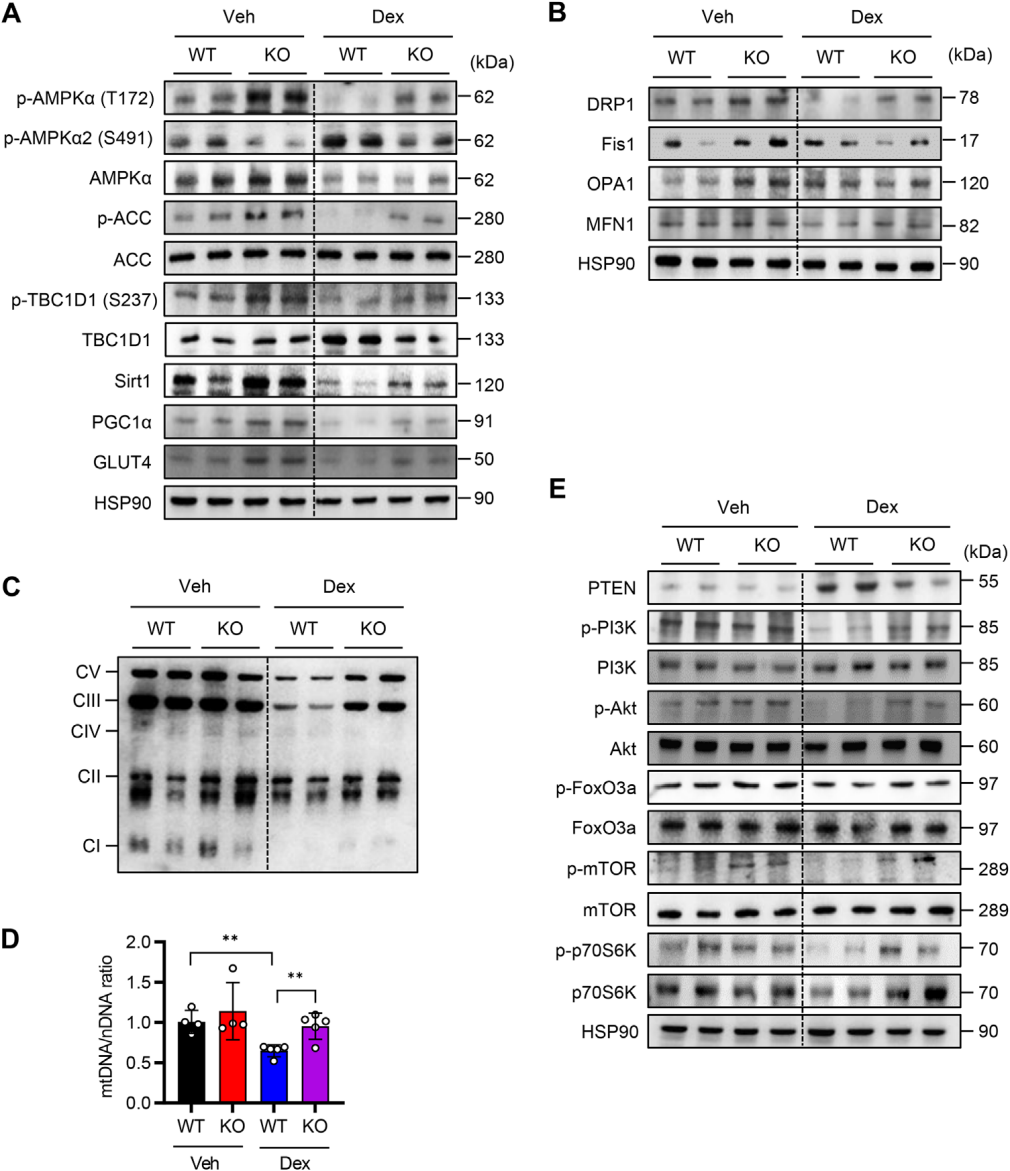
Activation of AMPKα and enhancement of mitochondrial biogenesis by PAK4 deficiency. (A–C) Protein levels of AMPKα signaling (A), mitochondrial dynamics (B), and OxPhos complex (C) in gastrocnemius muscles were analyzed by Western blotting. (D) Mitochondrial DNA (mtDNA) was quantified by qPCR using nuclear DNA (nDNA) as a reference. (E) Western blot analysis of the PI3K‐Akt pathway in gastrocnemius muscle. Values are mean ± SD (*n* = 4 for Veh, *n* = 5 for Dex). ^**^ *p *< 0.01.

Since dexamethasone treatment increased both PAK4 protein levels and AMPKα2 phosphorylation at S491 (Figure 1A), we further investigated the role of PAK4 in regulating AMPK signaling in atrophic muscles. In WT mice, dexamethasone treatment reduced phosphorylations of AMPKα‐T172 and its substrate ACC and TBC1D1, increased AMPKα2‐S491 phosphorylation, and decreased Sirt1 and PGC‐1α levels (Figure 3A; Figure S6A). However, these effects were attenuated in *Pak4* KO mice. Consistent with the elevated Sirt1 and PGC‐1α levels, *Pak4* KO mice also exhibited increased protein levels of mitochondrial pro‐fission protein dynamin‐related protein 1 (DRP1) [25], with no effect on other mitochondrial dynamics regulators such as fission 1 (Fis1), optic atrophy 1 (OPA1), or mitofusin 1 (MFN1) (Figure 3B; Figure S6B). Additionally, they displayed higher levels of electron transport chain proteins and increased mitochondrial DNA content compared with WT mice (Figure 3C,D; Figure S6C). In line with our previous study [23], PAK4 deficiency reduced PTEN expression, thereby activating the PI3K/Akt pathway (Figure 3E; Figure S6D). This activation also increased phosphorylation of mTOR, p70S6K, and FoxO3a, which may promote protein synthesis and suppress FoxO3a‐dependent protein degradation [26]. Taken together, these findings suggest that PAK4 deficiency enhances mitochondrial biogenesis and dynamics while reducing protein turnover, thereby protecting against dexamethasone‐induced muscle atrophy.

Previous studies have suggested that Akt phosphorylates AMPKα2 at S491 [27]. To further investigate the potential interplay between PAK4 and Akt in this process, we examined their effects on AMPKα2‐S491 phosphorylation. In C2C12 cells, PAK4 overexpression enhanced dexamethasone‐induced S491 phosphorylation while reducing Akt phosphorylation (Figure S7A). Moreover, insulin‐stimulated AMPKα2‐S491 phosphorylation was not diminished by the Akt inhibitor MK2206, despite complete suppression of Akt phosphorylation (Figure S7B). In contrast, our previous study showed that *Pak4* deletion attenuated insulin‐induced AMPKα1/2‐S485/491 phosphorylation [22]. Together, these findings indicate that PAK4 mediates AMPKα2‐S491 phosphorylation independently of Akt, whereas insulin‐induced phosphorylation appears to be partially dependent on PAK4.

**FIGURE 4:**
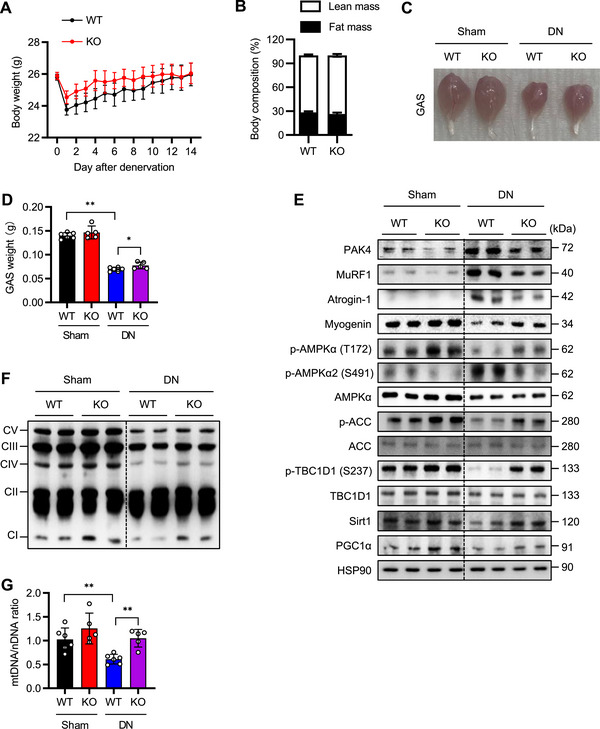
Mitigation of denervation (DN)‐induced muscle atrophy by PAK4 deficiency. (A, B) Eight‐week‐old male *Pak4* KO mice and their WT littermates underwent sciatic nerve transection (left limb) and sham surgery (right limb). Body weight change over time (A) and lean body mass (B) were monitored. (C, D) Representative images of gastrocnemius (GAS) muscle morphology (C) and wet weight (D). (E, F) Protein levels of PAK4, AMPKα, atrophy‐related genes (E) and OxPhos complex (F) in GAS muscles were analyzed by Western blotting. (G) Mitochondrial DNA (mtDNA) was quantified by qPCR, using nuclear DNA (nDNA) as a reference. Values are mean ± SD (*n* = 6 for WT, *n* = 5 for KO). ^*^ *p *< 0.05 and ^**^ *p *< 0.01.

We then investigated the impact of PAK4 deficiency in an alternative muscle atrophy model, sciatic nerve denervation. Consistent with findings from the dexamethasone‐induced muscle atrophy model, PAK4 deficiency effectively protected mice from muscle atrophy triggered by sciatic nerve denervation (Figure 4A–G; Figure S8A–E).

To assess the impact of PAK4 deficiency on muscle function, we conducted ex vivo measurements of isometric force and fatigue sensitivity. Gastrocnemius muscles from *Pak4* KO mice, whether exposed to dexamethasone or subjected to sciatic nerve transection, displayed greater tetanic force compared with WT controls, whereas twitch force remained comparable between groups (Figure S9A,B,D,E). Additionally, *Pak4* KO muscles exhibited higher fatigue resistance following dexamethasone treatment or nerve denervation (Figure S9C,F).

### 2.3 Overexpression of Phospho‐Deficient Mutant of AMPKα2^S491A^ Prevents Dexamethasone‐Induced Muscle Atrophy

**FIGURE 5:**
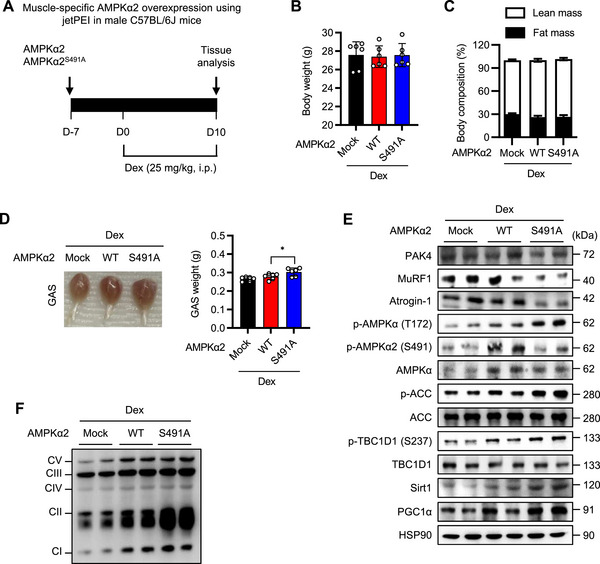
Attenuation of muscle atrophy by skeletal muscle‐specific overexpression of AMPKα2^S491A^. (A) Schematic diagram of the experimental protocols. Plasmids encoding wild‐type AMPKα2 (WT) or phospho‐deficient mutant of AMPKα2 (S491A) were injected into gastrocnemius (GAS) muscles of 11‐week‐old male C57BL/6J mice using jet‐PEI transfection. One week later, dexamethasone (25 mg/kg, i.p.) was administered daily for 10 days, and muscle atrophy was assessed. (B, C) Body weight change (B) and lean body mass (C) were measured. (D) Representative images of GAS muscle morphology and wet weight. (E, F) Western blot analysis of the AMPK pathways and OxPhos complex in GAS muscle. Values are mean ± SD (*n* = 6 per each group). ^*^ *p *< 0.05.

To determine the causal relationship between AMPKα2‐S491 phosphorylation and PAK4‐mediated muscle atrophy, we investigated the effects of an intramuscular injection of a plasmid encoding a phospho‐deficient mutant AMPKα2^S491A^ using the jet‐PEI technique as described previously [22] (Figure 5A). Overexpression of either wild‐type AMPKα2 (WT) or AMPKα2^S491A^ (S491A) had no impact on body weight, food intake, or lean body mass composition in dexamethasone‐treated mice (Figure 5B,C; Figure S10A). PAK4 protein levels did not differ among groups, suggesting PAK4 does not act downstream of AMPK. However, AMPKα2^S491A^ attenuated atrophy, as evidenced by increased gastrocnemius muscle mass, reduced MuRF1 and Atrogin‐1 expression, enhanced AMPKα activity, and elevated mitochondrial OxPhos proteins (Figure 5D–F; Figure S10B,C).

### 2.4 Pharmacological Degradation of PAK4 Alleviates Muscle Atrophy

**FIGURE 6:**
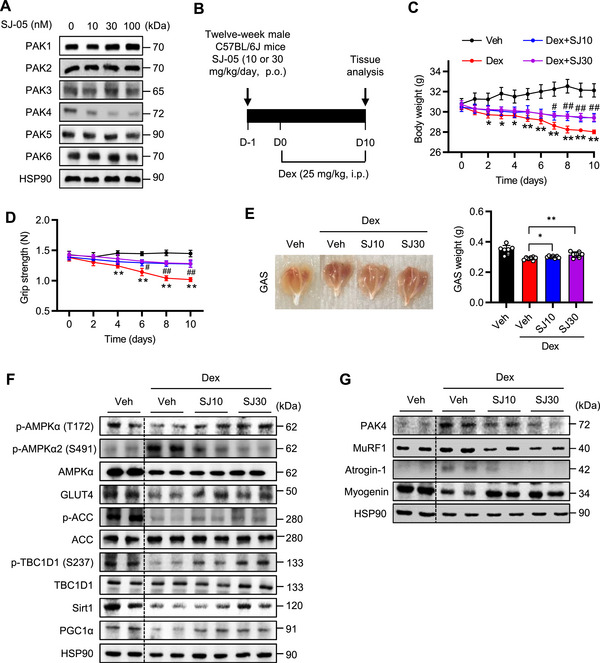
Mitigation of dexamethasone (Dex)‐induced muscle atrophy by PAK4 proteolysis‐targeting chimera (PROTAC). (A) C2C12 myotubes were treated with indicated concentrations of PAK4 PROTAC SJ‐05 for 24 h, and protein levels of PAK isozymes were analyzed by Western blotting. (B) Schematic representation of the experimental protocols. Twelve‐week‐old male C57BL/6J mice were administered SJ‐05 (10 or 30 mg/kg) daily for 10 consecutive days, starting one day before dexamethasone administration. Dexamethasone was administered daily at a dose of 25 mg/kg (i.p.) for 10 days. (C, D) Body weight changes over time (C) and forelimb grip strength (D) were measured. ^*^ *p *< 0.05 and ^**^ *p *< 0.01 vs. Veh; ^#^ *p *< 0.05 and ^##^ *p *< 0.01 vs. Dex. (E) Representative images of gastrocnemius (GAS) muscle morphology and wet weight. ^*^ *p* < 0.05 and ^**^ *p* < 0.01. (F, G) Protein levels of AMPKα signaling (F) and atrophy‐related proteins in GAS muscles were analyzed by Western blotting. Values are mean ± SD (*n* = 7).

The PROTAC‐based PAK4 degrader, SJ‐05, is a heterobifunctional small molecule designed to target PAK4 for degradation by linking a PAK4‐binding ligand to the E3 ligase cereblon (CRBN) via a chemical linker [17]. Treatment with SJ‐05 selectively reduced PAK4 protein levels in C2C12 cells (Figure 6A; Figure S11A). Consistent with findings from *Pak4* KO mice (Figure 2), daily oral administration of SJ‐05 (10 or 30 mg/kg) for 10 days increased body weight and grip strength without affecting food intake compared with dexamethasone‐only treatment (Figure 6B–D; Figure S11B). Furthermore, SJ‐05 increased gastrocnemius muscle wet weight and myofiber size compared with dexamethasone‐only treatment (Figure 6E; Figure S11C). Western blot analysis and mitochondrial DNA quantification further demonstrated activation of the AMPK pathway, reduced levels of dexamethasone‐induced muscle atrophy‐related proteins, and enhanced mitochondrial biogenesis and dynamics following SJ‐05 treatment (Figure 6F,G; Figure S11D–H).

Similar protective effects against muscle atrophy by SJ‐05 were observed in dexamethasone‐treated C2C12 cells (Figure S12A,B). Seahorse analysis in C2C12 cells showed that dexamethasone suppressed mitochondrial respiration, which was slightly but significantly improved by SJ‐05 (30 nM) (Figure S12C).

As *Pak4* KO and PROTAC treatment consistently exhibited AMPK activation with repressed AMPKα2‐S491 phosphorylation and protection from muscle atrophy, we hypothesized that the PAK4–AMPK pathway could be the major regulator of muscle atrophy. To investigate the causal relationship between PAK4 inhibition‐mediated AMPK activation and muscle atrophy, C2C12 cells were transfected with a plasmid encoding the phospho‐mimetic mutant AMPKα2^S491D^ and treated with dexamethasone, with or without SJ‐05. In AMPKα2^S491D^‐transfected cells, SJ‐05‐induced activation of the AMPKα‐PGC1α pathway, as well as the associated improvements in myofiber size and mitochondrial respiration, were abolished (Figure S13A–C).

### 2.5 PAK4 Expression and AMPKα2‐S491 Phosphorylation Are Linked to Human Sarcopenia

**FIGURE 7:**
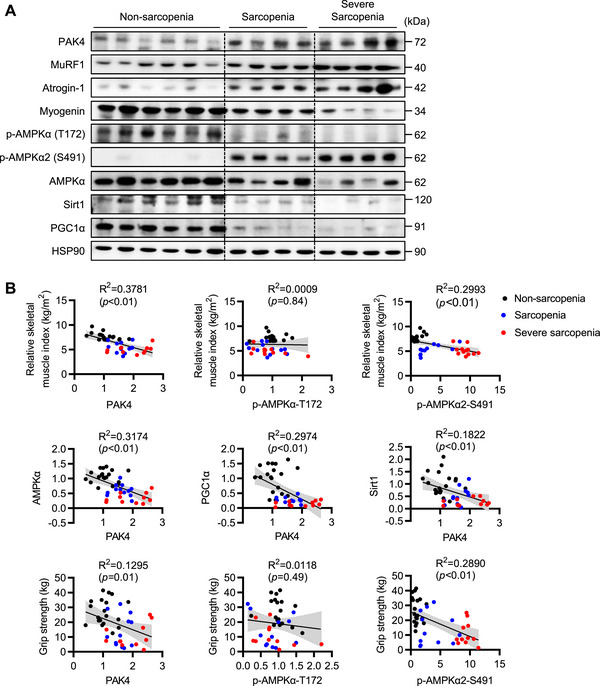
Relationship between PAK4 expression in skeletal muscle tissues and sarcopenic parameters in humans. (A) Representative Western blot analysis of PAK4 and p‐AMPKα‐S491 in tensor fascia lata muscle tissues. (B) Correlation between the PAK4 and its downstream protein levels and the sarcopenic parameters (*n* = 42). Values are mean ± SD.

Finally, to explore the potential role of PAK4 in human sarcopenia, we analyzed muscle samples from non‐sarcopenic individuals and sarcopenic patients across various age groups (Table S1). Compared with non‐sarcopenia controls, sarcopenic muscle tissues exhibited elevated PAK4 protein levels and increased phosphorylation of its downstream target, AMPKα2‐S491, along with upregulation of MuRF1 and Atrogin‐1 (Figure 7A; Figure S14A). In contrast, total AMPKα levels were reduced in sarcopenic muscle tissues.

Notably, PAK4 expression and AMPKα2‐S491 phosphorylation in muscle tissues were linked to sarcopenic parameters, showing an inverse relationship with grip strength, whereas AMPKα‐T172 phosphorylation showed no correlation with sarcopenia markers (Figure 7B). Additionally, the expression of Sirt1 and PGC‐1α was repressed in sarcopenic muscle tissues and positively correlated with AMPKα levels (Figure 7A; Figure S14B). These findings further highlight the role of PAK4 and AMPKα2‐S491 phosphorylation in the pathogenesis of sarcopenia.

## 3 Discussion

There is considerable interest in developing therapeutic approaches to prevent muscle atrophy. This study reveals that PAK4 protein is overexpressed and activated in various mouse and human models of sarcopenia. Oral PAK4 PROTAC treatment effectively prevented dexamethasone‐induced muscle atrophy in mice. Our findings suggest that targeting PAK4 with PROTAC could be a viable therapeutic strategy for sarcopenia, making this the first study to demonstrate PROTAC as a potential treatment for the condition. Additionally, selective deletion of PAK4 in skeletal muscle protected against muscle dysfunction in both dexamethasone‐ and denervation‐induced muscle atrophy. Mechanistically, extending our previous findings [22], we show that PAK4 inhibits AMPKα activity through inhibitory phosphorylation at S491 of AMPKα2 in sarcopenia models. As a result, mice lacking PAK4 or treated with PAK4 PROTAC display activation of the AMPKα–PGC1α pathway and enhanced mitochondrial biogenesis. In keeping with our previous works [22, 23], PAK4 deficiency also led to the activation of the PI3K/Akt pathway. Activation of Akt may phosphorylate and inhibit FoxO3, thereby repressing the transcription of atrogenes such as MuRF1 and Atrogin‐1, while promoting growth signals to enhance anabolic pathways (Graphical abstract). Therefore, PAK4 inhibitors could serve as a two‐in‐one strategy by enhancing anabolic signaling while reducing catabolic signaling in sarcopenia. Moreover, the clinical significance of the PAK4–AMPK pathway in sarcopenia is underscored by the finding that PAK4 protein levels and AMPKα2‐S491 phosphorylation were not only elevated but also negatively correlated with the skeletal muscle mass and grip strength in muscle tissues from sarcopenic patients compared with non‐sarcopenic individuals.

Muscle atrophy induced by dexamethasone can occur as a side effect of high‐dose or prolonged glucocorticoid treatment for chronic inflammatory diseases, increasing the risk of falls and impairing quality of life. Furthermore, fasting or caloric restriction stimulates the hypothalamus–pituitary–adrenal axis to increase glucocorticoid secretion and GR‐mediated muscle atrophy [28, 29]. Dexamethasone induces muscle atrophy through multiple pathways: it promotes the transcription of the key ubiquitin ligases MuRF1 and Atrogin‐1, enhances protein degradation, inhibits protein synthesis, and induces myostatin [30, 31, 32]. We observed that both the mRNA and protein levels of PAK4 were elevated following dexamethasone treatment. This upregulation is partly attributed to transcriptional activation, as the cis‐regulatory element GRE is present in the *Pak4* promoter, which was confirmed by the elevation of *Pak4*‐promoter luciferase activity and mRNA levels. Thus, the dexamethasone–GR pathway induces the expression of both muscle atrophy‐related ubiquitin ligases and PAK4, with the latter exacerbating dexamethasone‐induced muscle dysfunction, thereby creating a feed‐forward cycle between dexamethasone and PAK4. Additionally, post‐transcriptional regulation appears to contribute to PAK4 protein upregulation, as PKA and Sirt1‒two negative regulators of PAK4 protein stability [19, 20]‒were downregulated in dexamethasone‐treated muscle and other atrophy models. These findings suggest that impaired Sirt1‐mediated deacetylation and PKA‐mediated phosphorylation may enhance PAK4 protein stabilization in sarcopenia. Other endogenous or environmental factors may also regulate PAK4 expression. Hypoxia, a known risk factor for muscle weakening in individuals living at high altitudes or with chronic diseases such as heart failure or peripheral arterial disease, contributes to muscle atrophy [33]. Previously, we demonstrated that hypoxia stimulates PAK4 transcription through hypoxia‐inducible factor‐1α in a liver and kidney ischemia‐reperfusion model in both mice and humans [17, 18]. Taken together, our findings suggest that PAK4 overexpression may be a key aggravating factor in pathological muscle loss and weakness.

While AMPK's role in maintaining cellular energy balance and homeostasis is well established, its function in muscle sarcopenia remains unclear. Notably, pharmacological AMPK activators, AICAR and metformin, have demonstrated opposing effects on skeletal muscle mass regulation. AICAR has been shown to protect against sarcopenia [8, 9], whereas metformin either failed to preserve muscle mass or induced muscle atrophy by upregulating myostatin [9, 34, 35]. Similarly, genetic models have reported conflicting results. In skeletal muscle‐specific inducible AMPKα1/α2 KO mice, loss of AMPK activity led to muscle weakness, glycogen depletion, and fibrosis during disuse atrophy [36], underscoring its critical role in maintaining muscle function. AMPKα has also been shown to prevent cancer‐induced metabolic dysfunction in mice [36]. However, contradictory findings suggest that AMPKα2 deficiency protects against denervation‐induced skeletal muscle atrophy [37]. Furthermore, little is known about the specific role of AMPKα phosphorylation in muscle function. Our study provides several key insights into AMPKα, particularly the phosphorylation of AMPKα2‐S491. First of all, AMPKα protein levels and its T172 phosphorylation were reduced in four different muscle atrophy mouse models and in human sarcopenia compared with respective controls. In contrast, AMPKα2‐S491 phosphorylation was elevated in individuals with muscle loss and correlated with increased PAK4 levels or the severity of sarcopenia. Notably, our findings on the reciprocal regulation of AMPKα2‐S491 and T172 phosphorylation in sarcopenia patients align with our previous observation that PAK4‐mediated AMPKα2‐S491 phosphorylation was elevated in the muscles of type 2 diabetic patients, while AMPKα‐T172 phosphorylation was suppressed [22]. This is also consistent with another report showing increased AMPK‐S485/491 phosphorylation in colorectal cancer, which inversely correlated with T172 phosphorylation [38]. Across these conditions, the level of AMPKα2‐S491 phosphorylation positively correlated with disease severity, highlighting its regulatory role in AMPK signaling. Both phosphorylation sites (S485/491) are located in the ST loop of the AMPKα subunit's C‐terminal region, which is extensively phosphorylated by multiple kinases. Phosphorylation at S485/491 enables the ST loop to interact with the minor lobe of the kinase domain, thereby blocking T172 phosphorylation [27]. Additionally, AMPKα‐S485/S491 phosphorylation has been reported to promote AMPKα degradation via E3 ubiquitin ligase‐mediated proteolysis [39]. Further research is needed to investigate the mechanisms underlying AMPKα downregulation in sarcopenia.

Secondly, acute ectopic overexpression of a phospho‐deficient AMPKα2 mutant (AMPKα2^S491A^) in muscle enhanced muscle mass and mitochondrial function in dexamethasone‐induced sarcopenic mice. This was accompanied by AMPK activation and increased PGC‐1α expression. Conversely, deletion or PROTAC‐mediated degradation of PAK4 restored AMPKα activity and mitochondrial function. How does AMPK positively regulate muscle function? We propose that the AMPKα–PGC‐1α pathway downstream of PAK4 plays a central role. PGC‐1α protein levels were downregulated in both mouse models of muscle atrophy but were restored by PAK4 inhibition (via genetic KO or PROTAC). Moreover, ectopic overexpression of AMPKα2 mutants, AMPKα2^S491A^ and AMPKα2^S491D^, respectively, increased and decreased PGC‐1α expression, regardless of PAK4 manipulation. These findings confirm that AMPKα acts downstream of PAK4 to regulate PGC‐1α expression, consistent with previous reports identifying AMPK as a key activator of PGC‐1α [40]. PGC‐1α is a crucial regulator of mitochondrial content and oxidative metabolism. Transgenic mice overexpressing PGC‐1α in glycolytic muscles exhibit enhanced mitochondrial content and oxidative metabolism [41], protecting against muscle atrophy induced by denervation, fasting [42], or aging [43]. In contrast, PGC‐1α KO in skeletal muscle leads to mitochondrial dysfunction without affecting fiber type determination [44]. Although metformin induces muscle atrophy through FoxO3 [34, 45, 46], our findings in human sarcopenic muscle support the idea that the AMPKα–PGC‐1α pathway is essential for maintaining muscle function. Collectively, these results suggest that AMPKα activity is reduced in sarcopenia and that targeting PAK4 may serve as a potential strategy to restore AMPKα activity and mitochondrial function.

We recently developed and validated the in vivo efficacy of the PAK4‐targeting PROTAC SJ‐05 in a mouse model of kidney ischemia‐reperfusion injury [17]. The current study confirmed that SJ‐05 effectively reduces PAK4 protein levels in the skeletal muscle of mice with muscle atrophy, highlighting its therapeutic potential. PROTAC technology is emerging as a promising therapeutic approach, as it enables the selective degradation of previously undruggable proteins rather than merely inhibiting their activity. Additionally, PROTACs can achieve efficacy at lower doses, minimizing potential toxicity. Unlike traditional inhibitors, which may have off‐target effects, PROTACs offer greater specificity by selectively degrading the target protein. To the best of our knowledge, this study is the first to demonstrate the therapeutic potential of PROTAC technology for treating muscle atrophy. Future studies are needed to further optimize SJ‐05 for enhanced efficacy and pharmacokinetic properties.

The limitations of this study are as follows: All experiments were performed exclusively in male mice, despite recent studies indicating potential sex‐specific differences in phenotype and molecular characteristics [47, 48]. Furthermore, we did not directly compare the therapeutic efficacy of PAK4 enzyme inhibitors with that of PROTACs, leaving the question of which approach is superior unanswered. Finally, the small sample size of human samples limits the clinical relevance of our findings. Addressing these issues will require further investigation.

## 4 Materials and Methods

### 4.1 Human Tissue Samples

Between July 2023 and April 2024, skeletal muscle specimens were collected from patients undergoing total hip arthroplasty in the Department of Orthopedic Surgery at Jeonbuk National University Hospital (Jeonju, Korea). In total, 42 samples were obtained: 19 from individuals without sarcopenia, 12 from patients with sarcopenia, and 11 from patients with severe sarcopenia (Table S1). Sarcopenia was diagnosed based on combined evaluations of muscle strength, physical performance, and muscle mass. Grip strength was measured using a Jamar dynamometer (Patterson Medical, Green Bay, WI, USA), and gait speed tests assessed functional performance. Muscle mass was quantified as the relative skeletal muscle index (RSMI) using dual‐energy X‐ray absorptiometry (Prodigy Fuga, GE Healthcare, Chicago, IL, USA) according to the Baumgartner equation [49]. Sarcopenia was defined as an RSMI below the established cutoffs (men: 7 kg/m^2^; women: 5.4 kg/m^2^) in combination with either reduced gait speed (<1.0 m/s) or decreased grip strength (men: <28 kg; women: <18 kg) [50]. Severe sarcopenia was diagnosed when RSMI, gait speed, and grip strength all fell below these cutoffs. During the direct anterior approach for total hip arthroplasty, tissue samples from the tensor fascia lata muscle were collected for Western blot analyses. Samples were immediately frozen and stored at −80°C. Written informed consent was obtained from all participants, and the study protocol was approved by the Institutional Review Board of Jeonbuk National University Hospital (Permit No. JBUH 2022‐11‐039), in accordance with the Declaration of Helsinki.

### 4.2 Animals

Skeletal muscle‐specific *Pak4* KO mice (*Pak4^flox/flox^*;*Myl1‐Cre*) were generated by crossing *Pak4^flox/flox^* mice (#015828, *B6.129S2‐Pak4^2.1Amin^/J*) with *Myl1‐Cre* mice (#024713, *Myl1^1(cre)sjb^/J*), both obtained from Jackson Laboratory (Bar Harbor, ME, USA), as previously described [22]. Mice were maintained in a controlled barrier facility at 23 ± 1°C, 60%–70% humidity, and a 12 h light/dark cycle.

For the dexamethasone‐induced muscle atrophy model, 12‐week‐old male *Pak4* KO mice and their wild‐type (WT, *Pak4^flox/flox^*) littermates received daily intraperitoneal injections of dexamethasone (25 mg/kg in saline) for 10 days. Body weight was monitored daily, grip strength measured with a grip strength meter (Jeung Do Bio & Plant, Seoul, South Korea), and body composition (lean and fat mass) assessed using a Bruker Minispec mq 7.5 NMR analyzer (Bruker Optics, Ettlingen, Germany). Unless otherwise noted, mice were euthanized 24 h after the last injection for tissue collection.

For the denervation‐induced muscle atrophy model, 8‐week‐old male WT and *Pak4* KO mice underwent unilateral sciatic nerve transection on the left limb, with sham surgery on the contralateral side. Fourteen days postdenervation, gastrocnemius muscles were excised under isoflurane anesthesia, weighed, and snap‐frozen in liquid nitrogen.

In the hindlimb unloading (disuse atrophy) model, the tails of 8‐week‐old male C57BL/6J mice were secured to a suspension apparatus in custom cages (15 × 26 × 55 cm) that allowed full 360° rotation while preventing hindlimb weight‐bearing, maintained for 14 days. Age‐matched mice housed normally served as controls.

For aging‐associated muscle atrophy, 22‐month‐old male C57BL/6J mice were compared with 2‐month‐old young controls (both from Samtako Bio Korea, Osan, Korea).

In the cancer cachexia model, 8‐week‐old male C57BL/6J mice received subcutaneous injections of 1 × 10^6^ Lewis lung carcinoma LLC1 cells (0.1 mL in saline) or saline alone into the upper flank. Animals were sacrificed 21 days postinjection for analysis.

For in vivo PAK4 overexpression experiments, 11‐week‐old male C57BL/6 mice received five intramuscular injections into each gastrocnemius (one proximal, three mid‐region, one distal), avoiding visible blood vessels. Each injection contained 2 × 10^9^ pfu of AdLacZ, AdPAK4, or AdPAK4^S474A^.

For in vivo AMPKα overexpression experiments, 11‐week‐old male C57BL/6 mice were injected intramuscularly into each gastrocnemius muscle (one proximal, three mid‐region, one distal) with 10 µg of mock, AMPKα2, or AMPKα2^S491A^ plasmid DNA in 22 µL of in vivo‐jetPEI reagent (#201‐05, Polyplus Transfection, New York, NY, USA) as previously described [22].

All procedures were approved by the Institutional Animal Care and Use Committees of the Korea Advanced Institute of Science and Technology (permit no. KA2024‐188‐v1) and Jeonbuk National University (permit no. JBNU 2023–112).

### 4.3 Cell Culture and Transient Transfection

Human embryonic kidney 293T cells (#CRL‐3216), mouse C2C12 myoblasts (#CRL‐1772), and Lewis lung carcinoma LLC1 cells (#CRL‐1642) were obtained from the American Type Culture Collection (Manassas, VA, USA). Cells were cultured in DMEM supplemented with 10% fetal bovine serum, 100 U/mL penicillin, and 100 µg/mL streptomycin at 37°C in a humidified atmosphere containing 5% CO_2_. Differentiation of C2C12 myoblasts was initiated at ∼80% confluence by switching to DMEM containing 2% horse serum, and fully formed myotubes were used for experiments after 4–6 days.

For *Pak4*‐promoter luciferase assays, cells were transiently transfected with 1 µg of *Pak4*‐ or GRE‐deleted *Pak4*‐luciferase plasmid. Luciferase activity was quantified using the Dual‐Luciferase Reporter Assay System (Promega, Madison, WI, USA) on a Lumat LB 9507 luminometer (Berthold, Bad Wildbad, Germany).

### 4.4 Mitochondrial Respiration

C2C12 myotubes were cultured for three days in DMEM, plated in XF24 microplates, and treated overnight with either vehicle (0.1% DMSO) or 30 nM SJ‐05. Oxygen consumption rates were measured using the Seahorse XF Cell Mito Stress Test Kit on an XF96 extracellular flux analyzer (Agilent Technologies, Santa Clara, CA, USA) according to manufacturer protocols [20]. Values were normalized to total protein content.

### 4.5 Statistics and Reproducibility

Data from at least three independent experiments with consistent results are presented as mean ± SD. Sample sizes were based on previous studies [51, 52]. Experiments were randomized and blinded, and all data were included. Normal distribution was assumed. Two‐group comparisons used unpaired Student's *t*‐tests; multi‐group comparisons used one‐way ANOVA or two‐way ANOVA with Tukey's post hoc test. Pearson correlation coefficients were calculated for continuous variables. *p* < 0.05 was considered significant. Analyses were performed using GraphPad Prism 9.5.

## Author Contributions

Jiacheng Du and Hwang Chan Yu carried out the in vitro and in vivo experiments. Young Jae Moon and Sun‐Jung Yoon collected and analyzed the human muscle tissue samples. Seung‐Yong Seo was responsible for designing and synthesizing the PAK4 PROTAC. Byung‐Hyun Park and Eun Ju Bae conceived the study, designed the experiments, interpreted the data, and drafted the manuscript. All authors critically reviewed and approved the final version of the manuscript.

## Funding

This research was supported by the Basic Science Research Program (2023R1A2C3002389) and the Bio & Medical Technology Development Program (RS‐2025‐02214748) through the National Research Foundation of Korea, by the Korea Health Technology R&D Project (HR22C1832) through the Korea Health Industry Development Institute, the Yuhan Innovation Program of Yuhan Corporation (awarded to B. H. P.), and the Glocal University 30 project at Jeonbuk National University in 2025 (awarded to E. J. B.).

## Ethics Statement

All animal procedures were approved by the Institutional Animal Care and Use Committees of the Korea Advanced Institute of Science and Technology (Permit No. KA2024‐188‐v1) and Jeonbuk National University (Permit No. JBNU 2023–112). All human participants provided written informed consent. The research was conducted in accordance with the Declaration of Helsinki, and the protocol was approved by the Institutional Review Board of Jeonbuk National University Hospital (Permit No. JBUH 2022‐11‐039).

## Conflicts of Interest

The authors declare no conflicts of interest.

## Permission to Reproduce Material from Other Sources

This study did not involve the use of any materials requiring reproduction permissions.

## Supporting information




**Figure S1: Western blotting for PAK family members**. Protein levels of PAK isotypes in gastrocnemius muscles: (A) 10 days after intraperitoneal injection of dexamethasone (Dex) at 25 mg/kg; (B) 21 days after subcutaneous implantation of LLC1 cells; (C) 14 days after hindlimb suspension (HS), and (D) in 2‐month‐old (young) and 22‐month‐old (old) C57BL/6J mice. Gastrocnemius muscles were removed for the analysis. Values are mean ± SD (*n* = 4 per group).
**Figure S2: Western blot analysis of AMPKα signaling pathways in sarcopenic mouse models**. Protein expression of AMPKα and its downstream signaling molecules was assessed in gastrocnemius muscles: (A) 10 days after intraperitoneal injection of dexamethasone (Dex, 25 mg/kg); (B) 14 days after hindlimb suspension (HS); (C) 21 days after subcutaneous implantation of LLC1 cells; and (D) in young (2‐month‐old) and aged (22‐month‐old) C57BL/6J mice. Protein band intensities were quantified. Data are presented as mean ± SD (*n* = 4 per group). ^*^ *p *< 0.05 and ^**^ *p *< 0.01.
**Figure S3: Regulation of PAK4 protein via Sirt1 and PKA pathways**. (A–D) Differentiated C2C12 myotubes were treated with 1 µM dexamethasone (Dex) for the indicated durations (A, B) or with varying concentrations of dexamethasone for 24 h (C, D), followed by analysis of PAK4 protein and mRNA levels (*n* = 3). (E) (Upper) Motif analysis of glucocorticoid receptor binding sites on the *Pak4* promoter based on predictions from the JASPAR database. (Lower) A schematic representation illustrating the mouse *Pak4* promoters. (F) GTEx analysis of human *PAK4* expression in relation to glucocorticoid receptor (GR) *NR3C1*. (G, H) HEK293T cells were transfected with either wild‐type *Pak4* (G) (*n* = 6) or GRE‐deleted *Pak4* mutants 1 and 2 (H) (*n* = 4), followed by a 24 h treatment with or without 1 µM dexamethasone. Subsequently, PAK4‐luciferase reporter activity was assessed (*n* = 6). (I–L) Analysis of Sirt1 expression and PKA activation in gastrocnemius muscles from mice under different conditions: vehicle vs. dexamethasone treatment (I), sham vs. hindlimb suspension (J), control vs. LLC1‐implanted cachexia (K), and young (2‐month‐old) vs. old (22‐month‐old) mice (L). Values are mean ± SD. ^*^, *p *< 0.05 and ^**^, *p *< 0.01.
**Figure S4: Mitigation of dexamethasone (Dex)‐induced muscle atrophy by PAK4 deficiency**. All experimental procedures were the same as those described in the Figure 2 legend. (A) Daily food intake was monitored in WT and KO mice over 10 days. (B) Myofiber cross‐sectional area corresponding to Figure 2E was quantified. (C) Western blot band intensities from Figure 2F were analyzed. (D) mRNA expression of *Trim63* (MuRF1) and *Fbxo32* (Atrogin‐1) in gastrocnemius muscle. (E–G) Representative images and wet weights of tibialis anterior (TA, E), extensor digitorum longus (EDL, F), and soleus (SOL, G) muscles. Data are presented as mean ± SD (*n* = 4 for Veh, *n* = 5 for Dex). ^*^ *p *< 0.05 and ^**^ *p *< 0.01.
**Figure S5: Aggravation of dexamethasone (Dex)‐induced muscle atrophy by PAK4 overexpression**. Adenovirus encoding wild‐type PAK4 (AdPAK4) or phospho‐deficient mutant of PAK4 (AdPAK4^S474A^) were overexpressed in gastrocnemius (GAS) muscles of 11‐week‐old male C57BL/6J mice. One week later, dexamethasone (25 mg/kg, i.p.) was administered daily for 10 days. Body weight was monitored every day (A). (B, C) Body weight at the end of the study (B) and daily food intake (C) were measured. (D) Representative images of gastrocnemius (GAS) muscle morphology and wet weight. (E, F) Western blot analysis of the AMPK pathways (E) and the OxPhos complex (F) in gastrocnemius muscle. (G) Immunofluorescence staining of laminin in muscle sections. Bar = 50 µm. Values are mean ± SD (*n* = 6 per group). ^**^ *p *< 0.01.
**Figure S6: Activation of AMPKα and enhancement of mitochondrial biogenesis by PAK4 deficiency**. Experimental procedures were identical to those described in the Figure 3 legend. (A–D) Quantification of Western blot band intensities corresponding to Figure 3A (A), 3B (B), 3D (C), and 3E (D). Data are shown as mean ± SD (*n* = 4 for Veh, *n* = 5 for Dex). ^*^ *p *< 0.05 and ^**^ *p *< 0.01.
**Figure S7: Effect of Akt inhibition on PAK4‐mediated AMPK phosphorylation**. (A) C2C12 myoblasts were infected with AdLacZ, AdPAK4, or AdPAK4^S474A^ and differentiated for 5 days, followed by dexamethasone (1 µM, 24 h) treatment. Phosphorylation of AMPK and Akt was assessed by Western blot. (B) PAK4‐overexpressing C2C12 cells were treated with Akt inhibitor MK2206 (5 µM, 24 h) and then stimulated with insulin (10 nM, 10 min). Phosphorylation of AMPK and Akt was analyzed by Western blot. Data are shown as mean ± SD (*n* = 3). ^*^, *p *< 0.05 and ^**^ *p *< 0.01.
**Figure S8: Mitigation of denervation (DN)‐induced muscle atrophy by PAK4 deficiency**. Experimental procedures were identical to those described in the Figure 4 legend. (A, B) Quantification of Western blot band intensities corresponding to Figure 4E (A) and 4F (B). (C–E) Representative images and wet weights of tibialis anterior (TA, C), extensor digitorum longus (EDL, D), and soleus (SOL, E) muscles. Data are presented as mean ± SD (*n* = 6 for WT, *n* = 5 for KO). ^*^ *p *< 0.05 and ^**^ *p *< 0.01.
**Figure S9: Enhanced force production and decreased muscle fatigue in gastrocnemius muscles from *Pak4* KO mice**. Gastrocnemius muscles were isolated from dexamethasone (Dex)‐ (A–C, *n* = 4 for Veh, *n* = 5 for Dex) and denervation (DN)‐induced atrophy mice (D–F, *n* = 3 per each group). (A, D) After mounting the gastrocnemius muscles on a force transducer, twitch force was measured by electrically stimulating the muscle with a single pulse (100 V for 1 ms). (B, E) The tetanic force–frequency relationships were determined by inducing contractions with incremental stimulation frequencies (1 ms pulses at 10–200 Hz for 500 ms at 100 V). (C, F) Fatigue index was measured at 1 Hz and 100 V using repeated stimuli for 7 min and expressed as a percentage of the initial contractile force. Values are mean ± SD. ^*^ *p *< 0.05 and ^**^ *p *< 0.01.
**Figure S10: Attenuation of muscle atrophy by skeletal muscle‐specific overexpression of AMPKα2^S491A^**. Experimental procedures were identical to those described in the Figure 5 legend. (A) Daily food intake. (B, C) Quantification of Western blot band intensities corresponding to Figure 3E (B) and 3F (C). Values are mean ± SD (*n* = 6 per group). ^*^ *p *< 0.05 and ^**^ *p *< 0.01.
**Figure S11: Mitigation of dexamethasone**‐**induced muscle atrophy by PAK4 PROTAC**. Experimental procedures were identical to those described in the Figure 6 legend. (A) Quantification of Western blot band intensities corresponding to Figure 6A (*n* = 3). SJ0, vehicle; SJ10, SJ 10 nM; SJ30, SJ 30 nM; SJ100, SJ, 100 nM. (B) Daily food intake (*n* = 7 per group). (C) Immunofluorescence staining of laminin in muscle sections. Myofiber cross‐sectional area was quantified (*n* = 7 per group). Bar = 50 µm. (D, E) Quantification of Western blot band intensities corresponding to Figure 6F (D) and 6G (E) (*n* = 7 per group). (F, G) Western blot analysis of mitochondrial dynamics (F) and OxPhos (G) proteins. (H) Mitochondrial DNA (mtDNA) was quantified by qPCR using nuclear DNA (nDNA) as a reference (*n* = 7 per group). Values are mean ± SD. ^*^ *p *< 0.05 and ^**^ *p *< 0.01.
**Figure S12: PAK4 PROTAC alleviates dexamethasone**‐**induced atrophic changes in C2C12 cells**. C2C12 cells were treated with 1 µM dexamethasone, with or without 30 nM SJ‐05 (SJ30) for 24 h. Atrogenes were analyzed by Western blotting (*n* = 3). (B) C2C12 cells were immunostained with anti‐laminin antibody, and myofiber diameter was quantified (*n* = 58–62). Bar = 50 µm. (C) The oxygen consumption rate (OCR) in C2C12 cells was measured using a Seahorse XF analyzer. Basal respiration, ATP production‐related respiration (uncoupled, calculated as the difference between OCR before and after oligomycin injection), and maximal respiration (difference between OCR after FCCP and after antimycin A (AA)/rotenone injection) were determined (*n* = 15). Values are mean ± SD. ^**^ *p *< 0.01.
**Figure S13: Reduction of SJ‐05 effects in C2C12 cells by AMPKα^S491D^ overexpression**. C2C12 cells were transfected with either AMPKα2 or MPKα2^S491D^ and treated with 1 µM dexamethasone, with or without 30 nM SJ‐05 (SJ30), for 24 h. (A) Activation of the AMPKα‐related pathway was analyzed by Western blotting (*n* = 3). (B) Cells were immunostained with anti‐MyHC antibody, and myofiber diameter was quantified (*n* = 60–68). Bar = 50 µm. (C) The oxygen consumption rate (OCR) in C2C12 cells was measured using a Seahorse XF analyzer (*n* = 9). Values are mean ± SD. ^*^ *p *< 0.05 and ^**^ *p *< 0.01.
**Figure S14: Relationship between key protein expressions in skeletal muscle tissues and sarcopenic parameters in humans**. Experimental procedures were identical to those described in the Figure 7 legend. (A) Quantification of Western blot band intensities corresponding to Figure 7A (*n* = 19 for nonsarcopenia, *n* = 12 for sarcopenia, *n* = 11 for severe sarcopenia). (B) Correlation between the PAK4 or AMPKα and the sarcopenic parameters, PGC1α, or Sirt1 (*n* = 42). Values are mean ± SD. ^*^ *p *< 0.05 and ^**^ *p *< 0.01. BMD, bone mineral density.
**Table S1**: Clinicopathologic variables in muscle donors.
**Table S2**: Antibodies used for western blotting, immunofluorescence, and immunohistochemical analyses.
**Table S3**: Information for primers (forward, FOR; reverse, REV).

## Data Availability

The datasets generated and analyzed during the current study are available from the corresponding author upon reasonable request.
