# Supplementary material for: Negative Impact of p21‐Activated Kinase 4‐Mediated AMP‐Activated Protein Kinase Inhibition on Sarcopenia in Mice and Humans
Source: MedComm (2020). 2025 Nov 29;6(12):e70508. doi: 10.1002/mco2.70508 (PMC12664904; doi:10.1002/mco2.70508)
Supplement: Supplementary file 1 — Figure S1: Western blotting for PAK family members. Protein levels of PAK isotypes in gastrocnemius muscles: (A) 10 days after intraperitoneal injection of dexamethasone (Dex) at 25 mg/kg; (B) 21 days after subcutaneous implantation of LLC1 cells; (C) 14 days after hindlimb suspension (HS), and (D) in 2‐month‐old (young) and 22‐month‐old (old) C57BL/6J mice. Gastrocnemius muscles were removed for the analysis. Values are mean ± SD (n = 4 per group). Figure S2: Western blot analysis of AMPKα signaling pathways in sarcopenic mouse models. Protein expression of AMPKα and its downstream signaling molecules was assessed in gastrocnemius muscles: (A) 10 days after intraperitoneal injection of dexamethasone (Dex, 25 mg/kg); (B) 14 days after hindlimb suspension (HS); (C) 21 days after subcutaneous implantation of LLC1 cells; and (D) in young (2‐month‐old) and aged (22‐month‐old) C57BL/6J mice. Protein band intensities were quantified. Data are presented as mean ± SD (n = 4 per group). * p < 0.05 and ** p < 0.01. Figure S3: Regulation of PAK4 protein via Sirt1 and PKA pathways. (A–D) Differentiated C2C12 myotubes were treated with 1 µM dexamethasone (Dex) for the indicated durations (A, B) or with varying concentrations of dexamethasone for 24 h (C, D), followed by analysis of PAK4 protein and mRNA levels (n = 3). (E) (Upper) Motif analysis of glucocorticoid receptor binding sites on the Pak4 promoter based on predictions from the JASPAR database. (Lower) A schematic representation illustrating the mouse Pak4 promoters. (F) GTEx analysis of human PAK4 expression in relation to glucocorticoid receptor (GR) NR3C1. (G, H) HEK293T cells were transfected with either wild‐type Pak4 (G) (n = 6) or GRE‐deleted Pak4 mutants 1 and 2 (H) (n = 4), followed by a 24 h treatment with or without 1 µM dexamethasone. Subsequently, PAK4‐luciferase reporter activity was assessed (n = 6). (I–L) Analysis of Sirt1 expression and PKA activation in gastrocnemius muscles from mice under different co [file MCO2-6-e70508-s001.pdf]

Negative impacts of p21-activated kinase 4-mediated AMP-activated protein kinase inhibition on sarcopenia in mice and humans

Supplementary Information

1. Supplementary Methods
2. Supplementary Figures
3. Supplementary Tables

1. Supplementary Methods

Ex vivo electrical pulse stimulation of skeletal muscle

Gastrocnemius muscles were isolated from euthanized mice and secured to stainless steel hooks using 6-0 nylon sutures attached to the tendons. Each muscle was mounted vertically between a force transducer (Model 159901, Radnoti, Monrovia, CA, USA) and an adjustable hook. The preparation was immersed in an organ bath containing O_2/CO_2 (95/5%)-bubbled Krebs–Ringer solution (4.75 mM KCl, 118 mM NaCl, 1.18 mM KH_2PO_4, 24.8 mM $NaHCO_3$, 1.18 mM $MgSO_4$, 2.5 mM $CaCl_2 \cdot 2H_2O$ and 10 mM glucose). Initial muscle length was adjusted to achieve maximal twitch force (100 V, 2 ms pulse). After equilibration, tetanic force was measured by applying 2 ms pulses at 10–200 Hz for 500 ms at 100 V, with 1-minute recovery intervals. Fatigue resistance was assessed by repetitive stimulation for 7 minutes at 1 Hz and 100 V. Force data were acquired and analyzed using LabChart Pro (Version 8, AD Instruments, Colorado Springs, CO, USA). At the conclusion of each experiment, muscle length and wet weight were recorded.

Histology

Freshly harvested gastrocnemius tissues were immersed in 30% sucrose, then embedded in liquid nitrogen–cooled isopentane. Cryosections (10 μm) were prepared and blocked with 5% goat serum for 40 minutes at room temperature. Sections were incubated overnight at 4°C with anti-laminin primary antibody (#L9393, Sigma-Aldrich, St. Louis, MO, USA), followed by Alexa Fluor 488–conjugated goat anti-rabbit IgG secondary antibody (#11008, Thermo Fisher Scientific, Waltham, MA, USA) for 40 minutes at room temperature. Nuclei were counterstained with DAPI. Images were captured using an LSM510 confocal microscope (Carl Zeiss, Oberkochen, Germany) and analyzed with iSolution DT 36 software (Carl Zeiss).

Western blotting

Equal amounts (20 μg) of protein from tissue homogenates or cell lysates were separated on 6–12% SDS–PAGE gels and transferred to PVDF membranes. After blocking with 5% skim milk, membranes were incubated with the appropriate primary antibodies (Table S2) and subsequently probed with HRP-conjugated secondary antibodies. Bands were visualized using a Las-4000 imaging system (GE Healthcare Life Sciences, Pittsburgh, PA, USA).

RNA isolation and qPCR

Total RNA was extracted from frozen skeletal muscle or differentiated C2C12 myotubes using an RNA Iso kit (TaKaRa, Tokyo, Japan). RNA was precipitated with isopropanol, washed in 70% ethanol, and resuspended in DEPC-treated distilled water. First-strand cDNA synthesis was performed using random hexamer primers with the First-Strand cDNA Synthesis Kit (Applied Biosystems, Foster City, CA, USA). Primers were designed using PrimerBank (https://pga.mgh.harvard.edu/primerbank; Table S3). qPCR reactions were carried out in 10 μl volumes containing 10 ng cDNA, 200 nM of each primer, and PCR master mix, in 384-well plates, using an ABI Prism 7900HT Sequence Detection System (Applied Biosystems).

2. Supplementary Figures

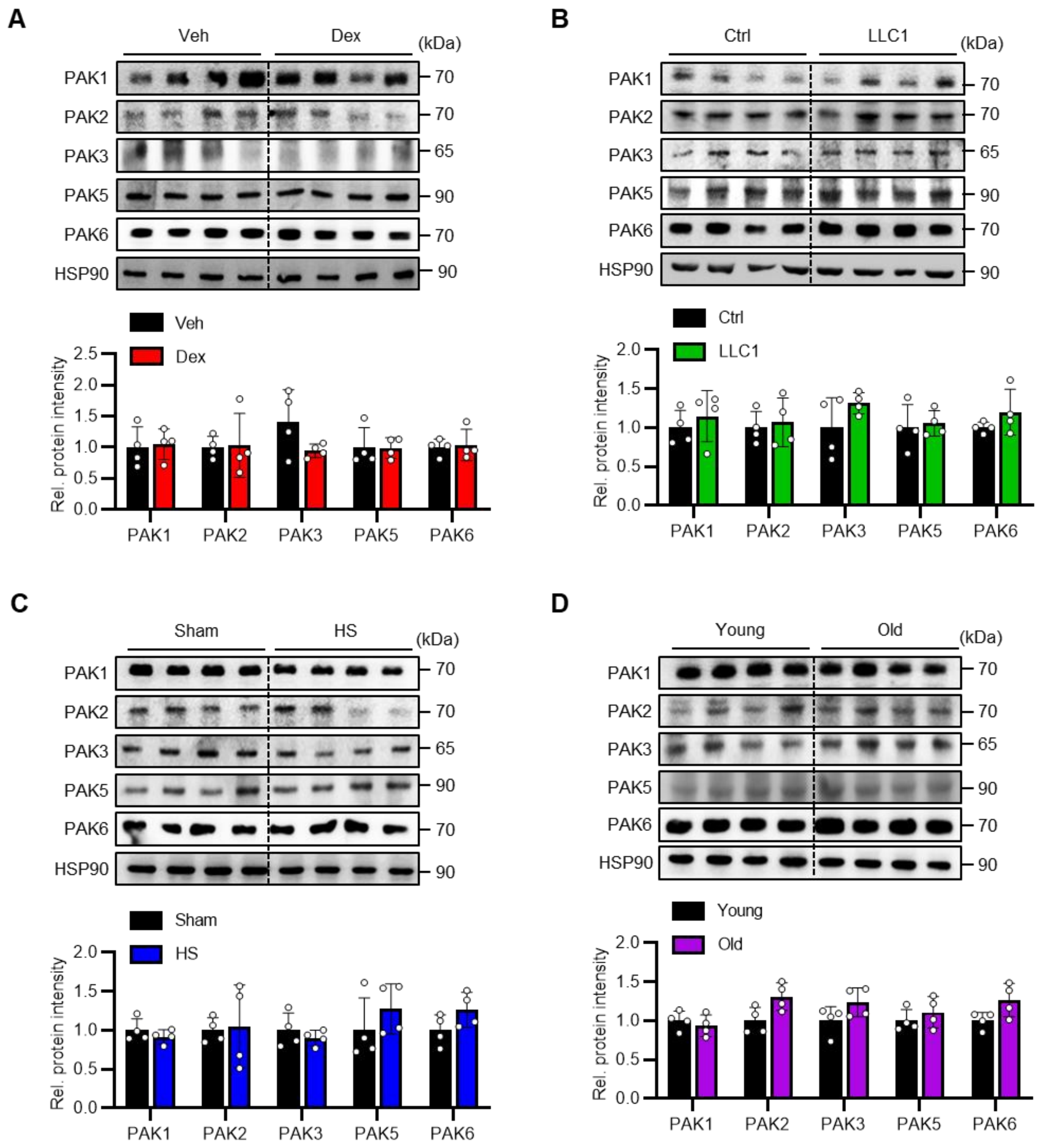

Figure S1. Western blotting for PAK family members. Protein levels of PAK isotypes in gastrocnemius muscles: (A) 10 days after intraperitoneal injection of dexamethasone (Dex) at 25 mg/kg, (B) 21 days after subcutaneous implantation of LLC1 cells, (C) 14 days after hindlimb suspension (HS), and (D) in 2-month-old (Young) and 22-month-old (Old) C57BL/6J mice. Gastrocnemius muscles were removed for the analysis. Values are mean ± SD (*n* = 4 per each group).

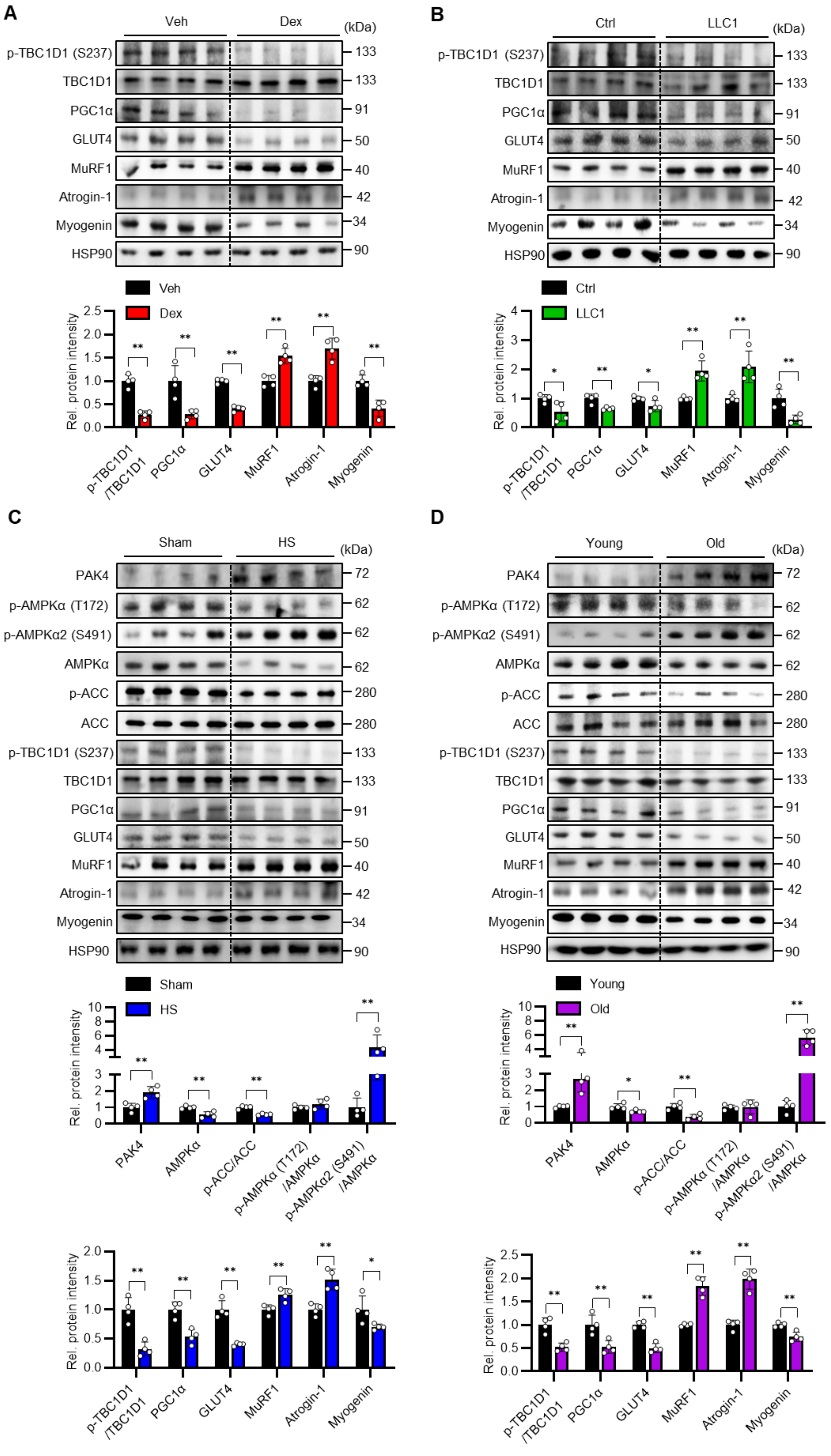
A
Veh
Dex
(kDa)
p-TBC1D1 (S237)
TBC1D1
PGC1α
GLUT4
MuRF1
Atrogin-1
Myogenin
HSP90
133
133
91
50
40
42
34
90
Veh
Dex
Rel. protein intensity
2.5
2.0
1.5
1.0
0.5
0.0
p-TBC1D1 /TBC1D1
PGC1α
GLUT4
MuRF1
Atrogin-1
Myogenin
B
Ctrl
LLC1
(kDa)
p-TBC1D1 (S237)
TBC1D1
PGC1α
GLUT4
MuRF1
Atrogin-1
Myogenin
HSP90
133
133
91
50
40
42
34
90
Ctrl
LLC1
Rel. protein intensity
4
3
2
1
0
p-TBC1D1 /TBC1D1
PGC1α
GLUT4
MuRF1
Atrogin-1
Myogenin
C
Sham
HS
(kDa)
PAK4
p-AMPKα (T172)
p-AMPKα2 (S491)
AMPKα
p-ACC
ACC
p-TBC1D1 (S237)
TBC1D1
PGC1α
GLUT4
MuRF1
Atrogin-1
Myogenin
HSP90
72
62
62
62
280
280
133
133
91
50
40
42
34
90
Sham
HS
Rel. protein intensity
10
8
6
4
3
2
1
0
PAK4
AMPKα
p-ACC/ACC
p-AMPKα (T172) /AMPKα
p-AMPKα2 (S491) /AMPKα
Rel. protein intensity
2.0
1.5
1.0
0.5
0.0
p-TBC1D1 /TBC1D1
PGC1α
GLUT4
MuRF1
Atrogin-1
Myogenin
D
Young
Old
(kDa)
PAK4
p-AMPKα (T172)
p-AMPKα2 (S491)
AMPKα
p-ACC
ACC
p-TBC1D1 (S237)
TBC1D1
PGC1α
GLUT4
MuRF1
Atrogin-1
Myogenin
HSP90
72
62
62
62
280
280
133
133
91
50
40
42
34
90
Young
Old
Rel. protein intensity
10
8
6
4
3
2
1
0
PAK4
AMPKα
p-ACC/ACC
p-AMPKα (T172) /AMPKα
p-AMPKα2 (S491) /AMPKα
Rel. protein intensity
2.5
2.0
1.5
1.0
0.5
0.0
p-TBC1D1 /TBC1D1
PGC1α
GLUT4
MuRF1
Atrogin-1
Myogenin

Figure S2. Western blot analysis of AMPKα signaling pathways in sarcopenic mouse models. Protein expression of AMPKα and its downstream signaling molecules was assessed in gastrocnemius muscles: (A) 10 days after intraperitoneal injection of dexamethasone (Dex, 25 mg/kg), (B) 14 days after hindlimb suspension (HS), (C) 21 days after subcutaneous implantation of LLC1 cells, and (D) in young (2-month-old) and aged (22-month-old) C57BL/6J mice. Protein band intensities were quantified. Data are presented as mean ± SD (*n* = 4 per group). *, $p<0.05$ and **, $p<0.01$.

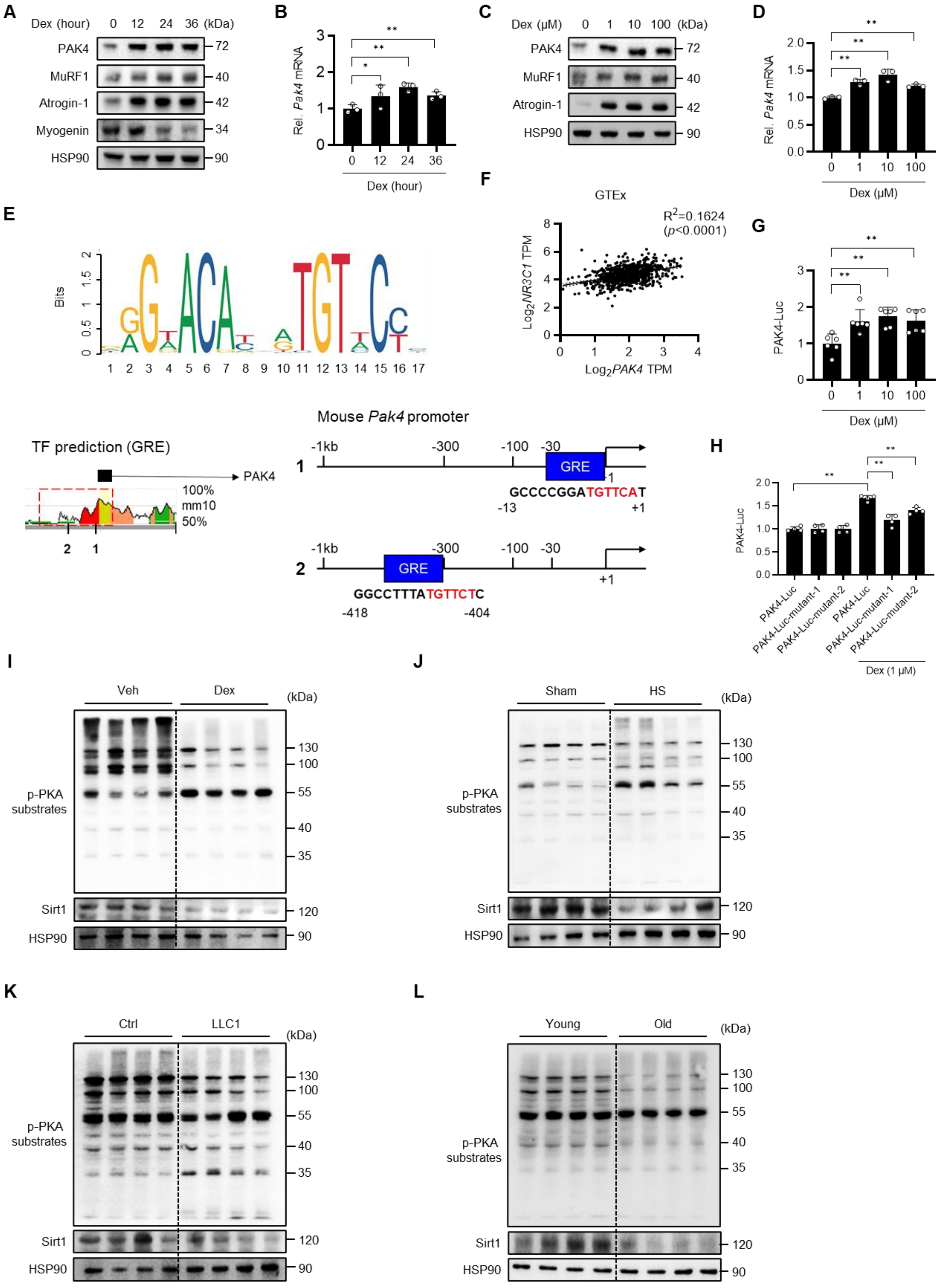

Figure S3. Regulation of PAK4 protein via Sirt1 and PKA pathways. (A-D) Differentiated C2C12 myotubes were treated with 1 μM dexamethasone (Dex) for the indicated durations (A, B) or with varying concentrations of dexamethasone for 24 hours (C, D), followed by analysis of PAK4 protein and mRNA levels (n = 3). (E) (Upper) Motif analysis of glucocorticoid

receptor binding sites on the *Pak4* promoter based on predictions from the JASPAR database. (Lower) A schematic representation illustrating the mouse *Pak4* promoters. (F) GTEx analysis of human *PAK4* expression in relation to glucocorticoid receptor (GR) *NR3C1*. (G, H) HEK293T cells were transfected with either wild-type *Pak4* (G) (n = 6) or GRE-deleted *Pak4* mutants 1 and 2 (H) (n = 4), followed by a 24-hour treatment with or without 1 μM dexamethasone. Subsequently, PAK4-luciferase reporter activity was assessed (n = 6). (I-L) Analysis of Sirt1 expression and PKA activation in gastrocnemius muscles from mice under different conditions: vehicle vs. dexamethasone treatment (I), sham vs. hindlimb suspension (J), control vs. LLC1-implanted cachexia (K), and young (2-month-old) vs. old (22-month-old) mice (L). Values are mean ± SD. *, p<0.05 and **, p<0.01.

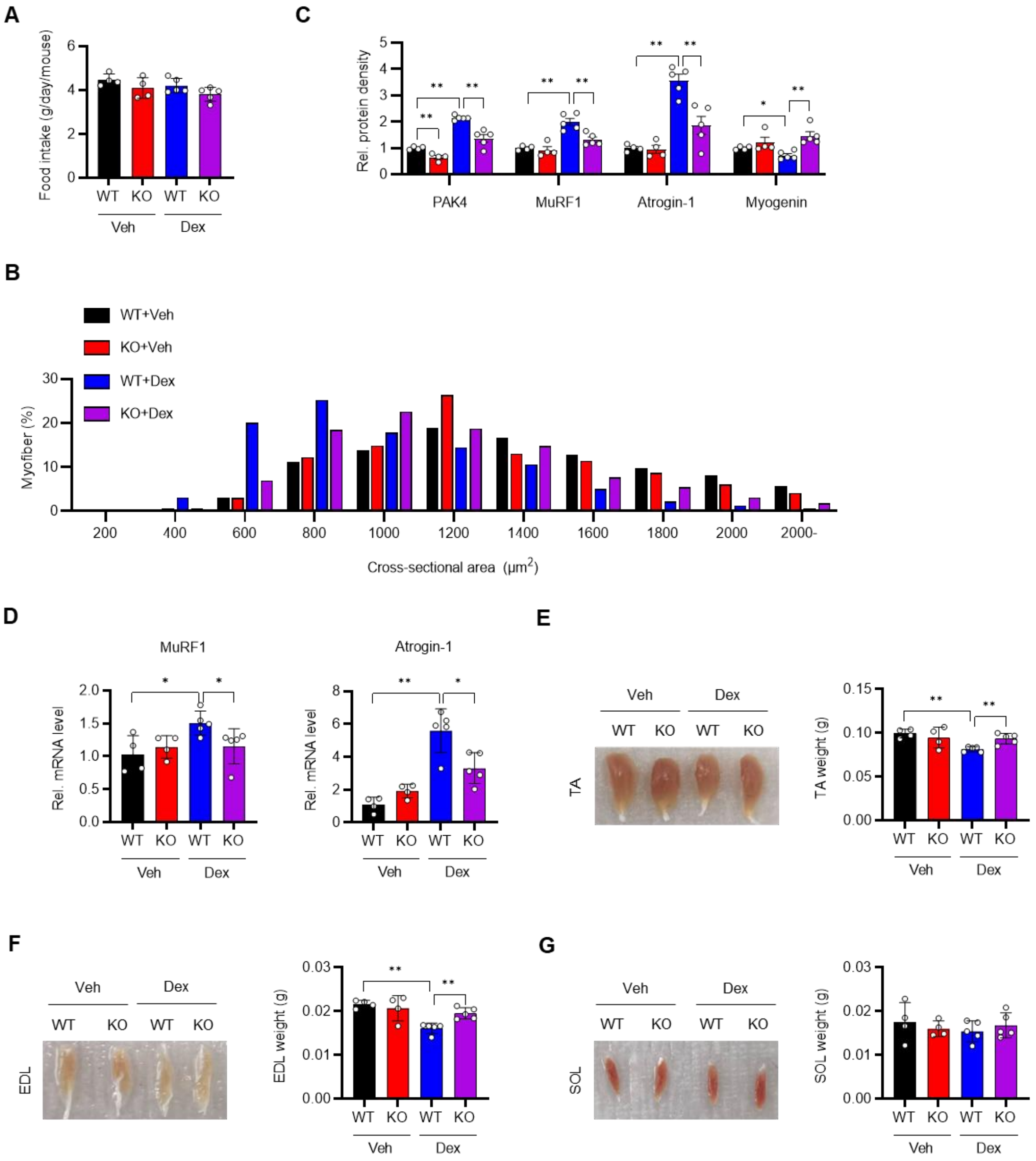

Figure S4. Mitigation of dexamethasone (Dex)-induced muscle atrophy by PAK4 deficiency. All experimental procedures were the same as those described in the Figure 2 legend. (A) Daily food intake was monitored in WT and KO mice over 10 days. (B) Myofiber cross-sectional area corresponding to Fig. 2E was quantified. (C) Western blot band intensities from Fig. 2F were analyzed. (D) mRNA expression of *Trim63* (MuRF1) and *Fbxo32* (Atrogin-1) in gastrocnemius muscle. (E–G) Representative images and wet weights of tibialis anterior (TA, E), extensor digitorum longus (EDL, F), and soleus (SOL, G) muscles. Data are presented as mean ± SD (n = 4 for Veh, n = 5 for Dex). *, p<0.05 and **, p<0.01.

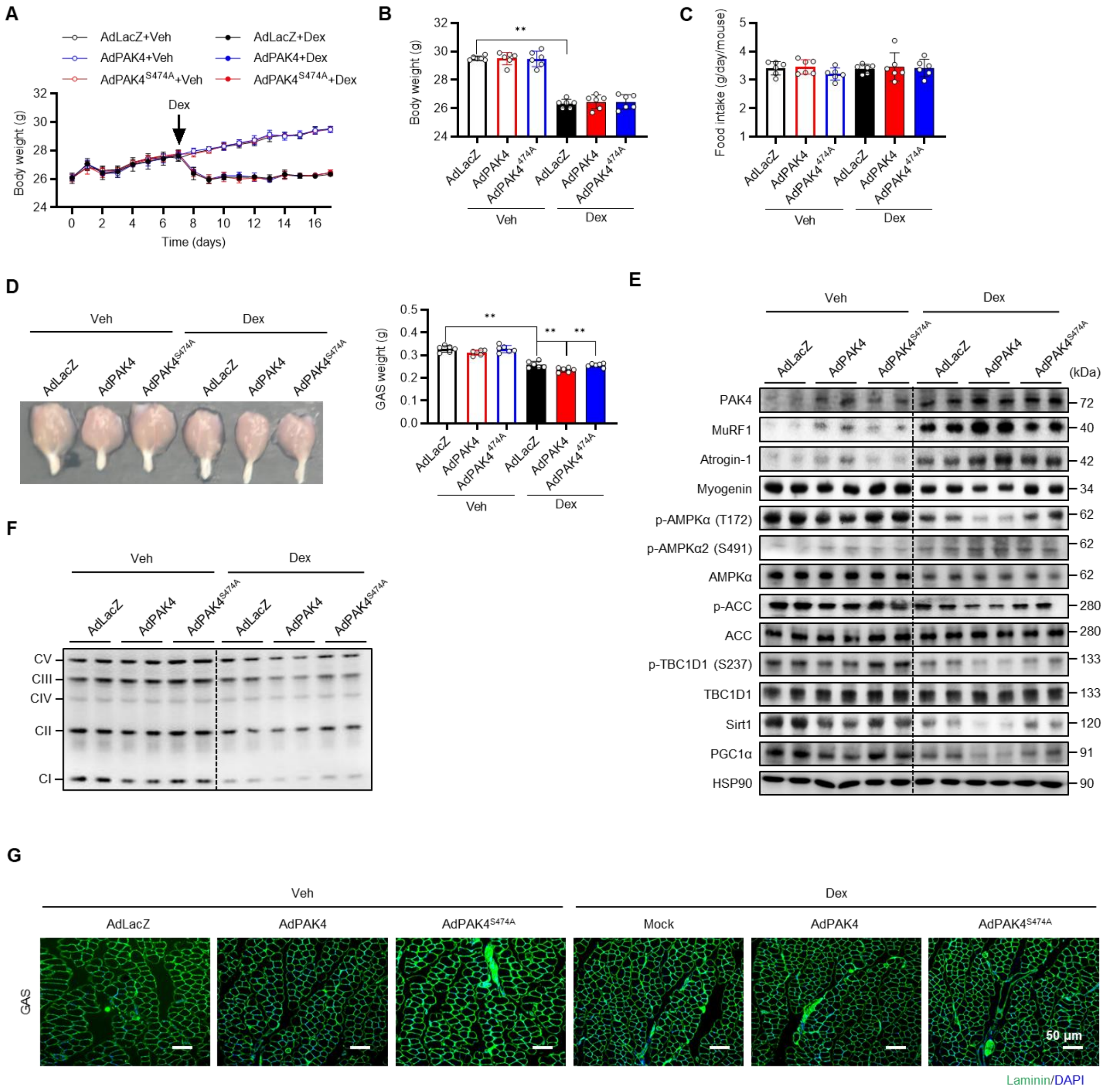

Figure S5. Aggravation of dexamethasone (Dex)-induced muscle atrophy by PAK4 overexpression. Adenovirus encoding wild-type PAK4 (AdPAK4) or phospho-deficient mutant of PAK4 (AdPAK4^{S474A}) were overexpressed in gastrocnemius (GAS) muscles of 11-week-old male C57BL/6J mice. One week later, dexamethasone (25 mg/kg, i.p.) was administered daily for 10 days. Body weight was monitored everyday (A). (B, C) Body weight at the end of the study (B) and daily food intake (C) were measured. (D) Representative images of gastrocnemius (GAS) muscle morphology and wet weight. (E, F) Western blot analysis of the AMPK pathways (E) and OxPhos complex (F) in gastrocnemius muscle. (G) Immunofluorescence staining of laminin in muscle sections. Bar=50 μm. Values are mean ± SD (*n* = 6 per each group). **, p<0.01.

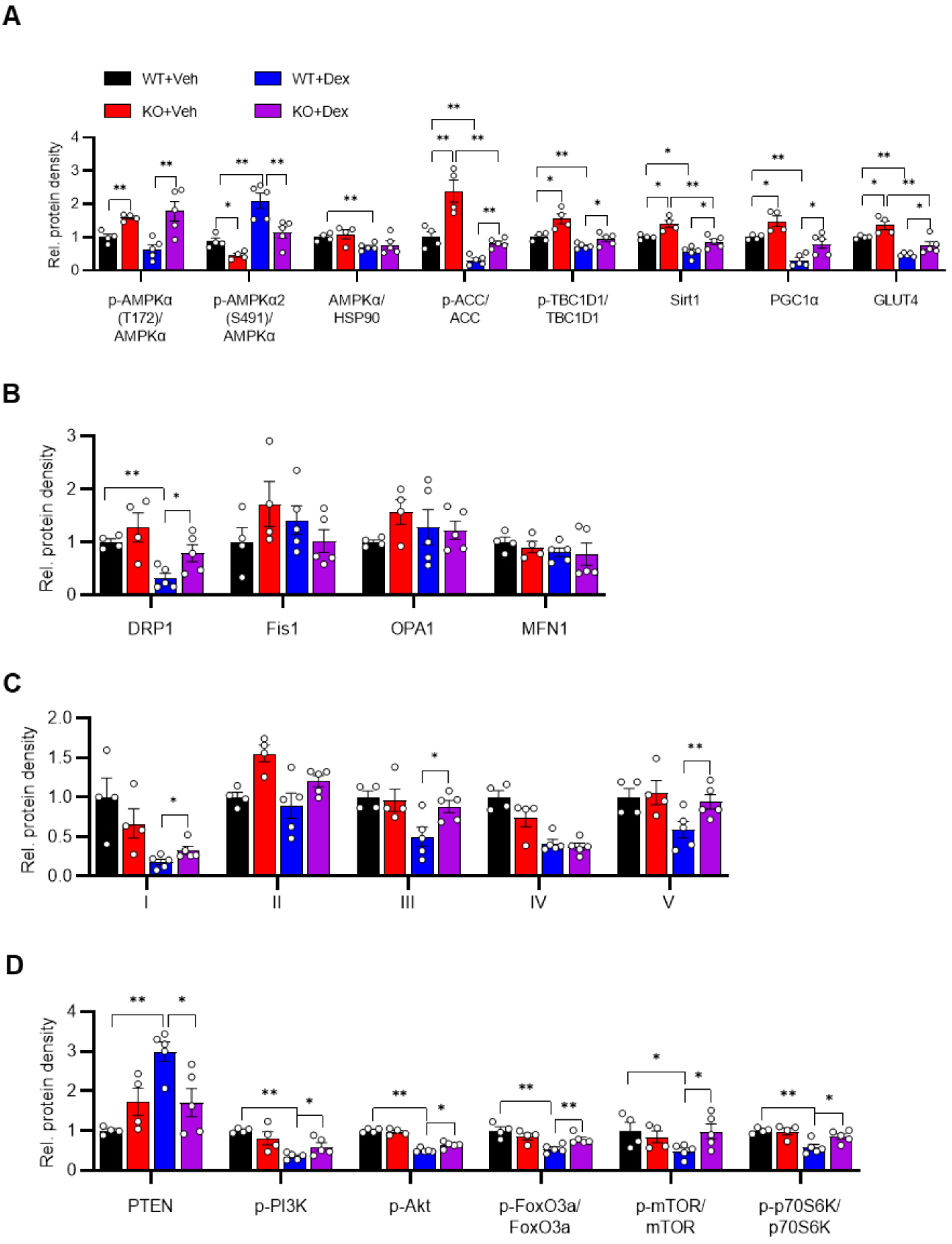

Figure S6. Activation of AMPKα and enhancement of mitochondrial biogenesis by PAK4 deficiency. Experimental procedures were identical to those described in the Figure 3 legend. (A–D) Quantification of Western blot band intensities corresponding to Fig. 3A (A), 3B (B), 3D (C), and 3E (D). Data are shown as mean ± SD (n = 4 for Veh, n = 5 for Dex). *, p<0.05 and **, p<0.01.

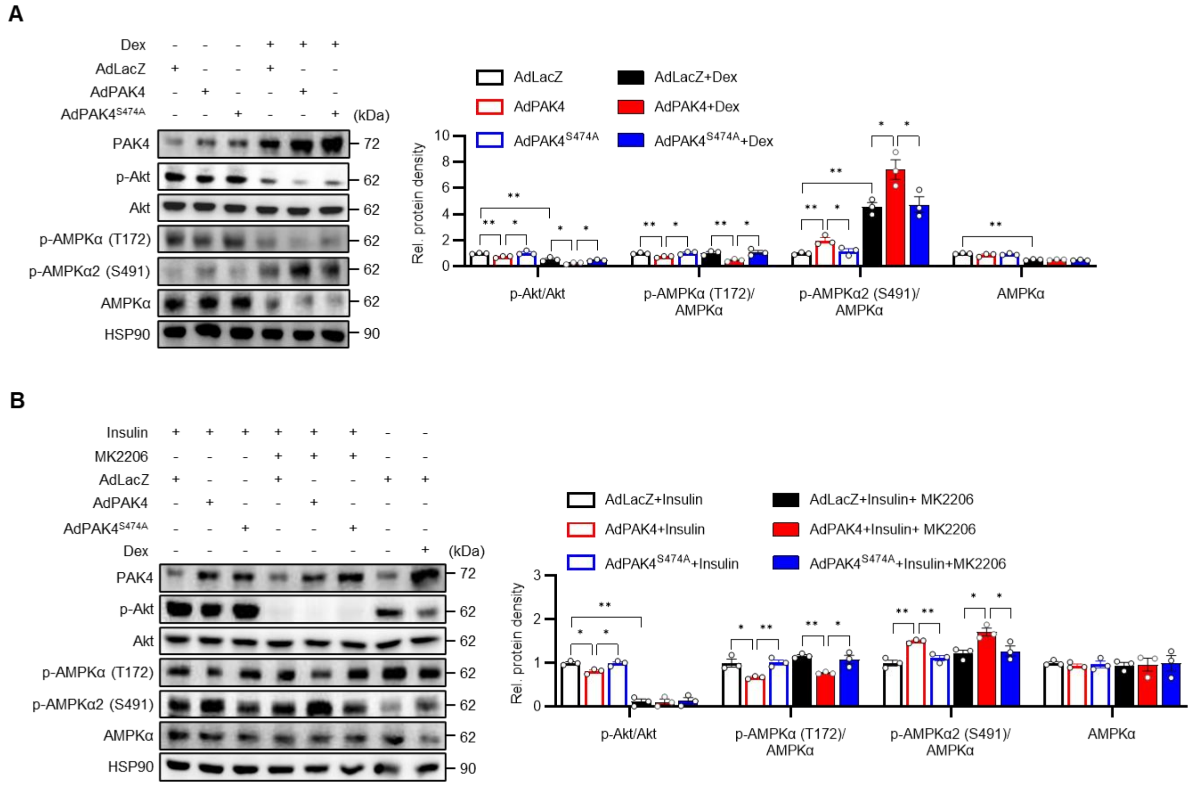

Figure S7. Effect of Akt inhibition on PAK4-mediated AMPK phosphorylation. (A) C2C12 myoblasts were infected with AdLacZ, AdPAK4 or AdPAK4^{S474A} and differentiated for 5 days, followed by dexamethasone (1 µM, 24 hours) treatment. Phosphorylation of AMPK and Akt was assessed by Western blot. (B) PAK4-overexpressing C2C12 cells were treated with Akt inhibitor MK2206 (5 µM, 24 hours) and then stimulated with insulin (10 nM, 10 minutes). Phosphorylation of AMPK and Akt was analyzed by Western blot. Data are shown as mean ± SD (n = 3). *, p<0.05 and **, p<0.01.

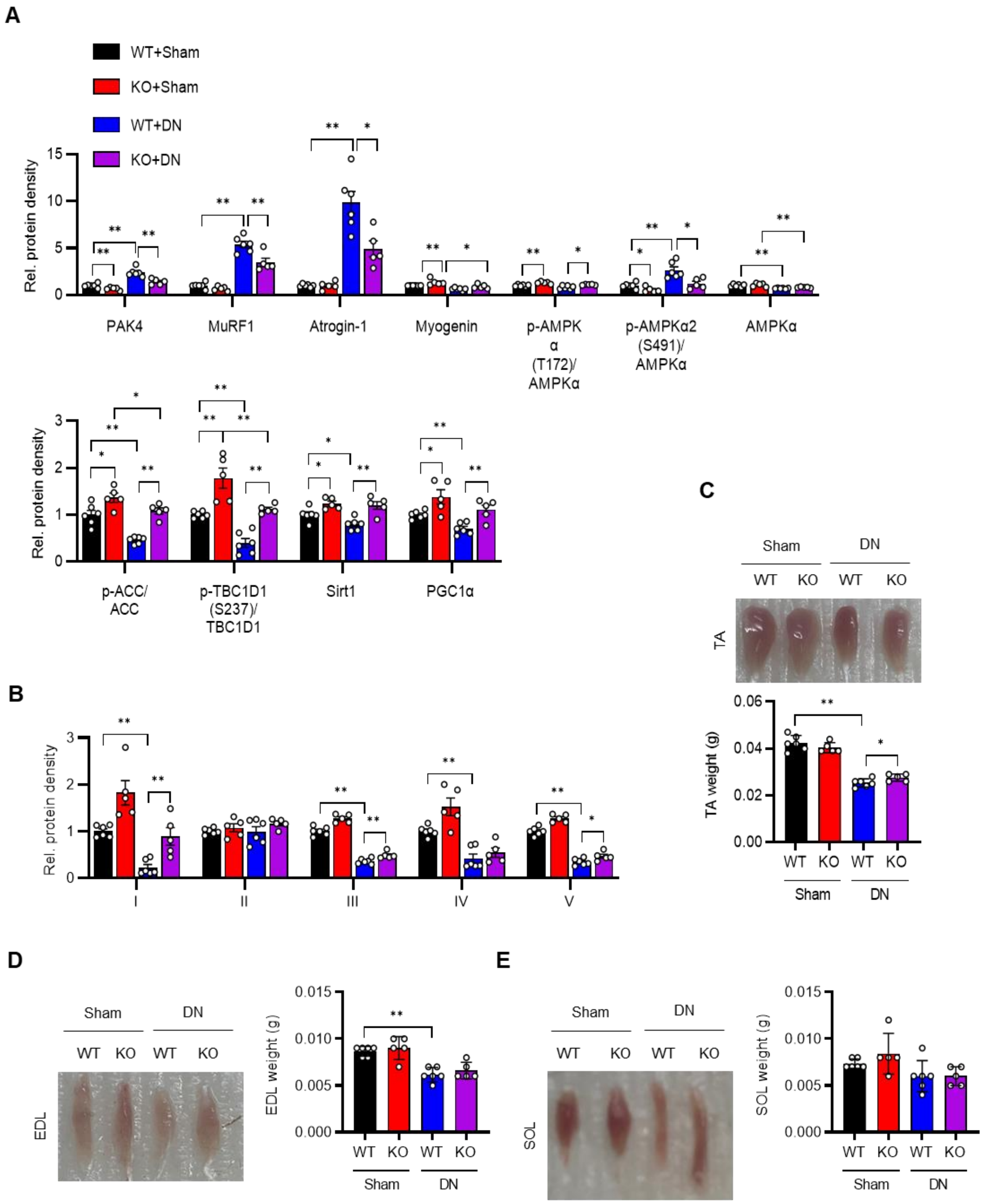

Figure S8. Mitigation of denervation (DN)-induced muscle atrophy by PAK4 deficiency. Experimental procedures were identical to those described in the Figure 4 legend. (A, B) Quantification of Western blot band intensities corresponding to Fig. 4E (A) and 4F (B). (C–E) Representative images and wet weights of tibialis anterior (TA, C), extensor digitorum longus (EDL, D), and soleus (SOL, E) muscles. Data are presented as mean ± SD (*n* = 6 for WT, *n* = 5 for KO). *, p<0.05 and **, p<0.01.

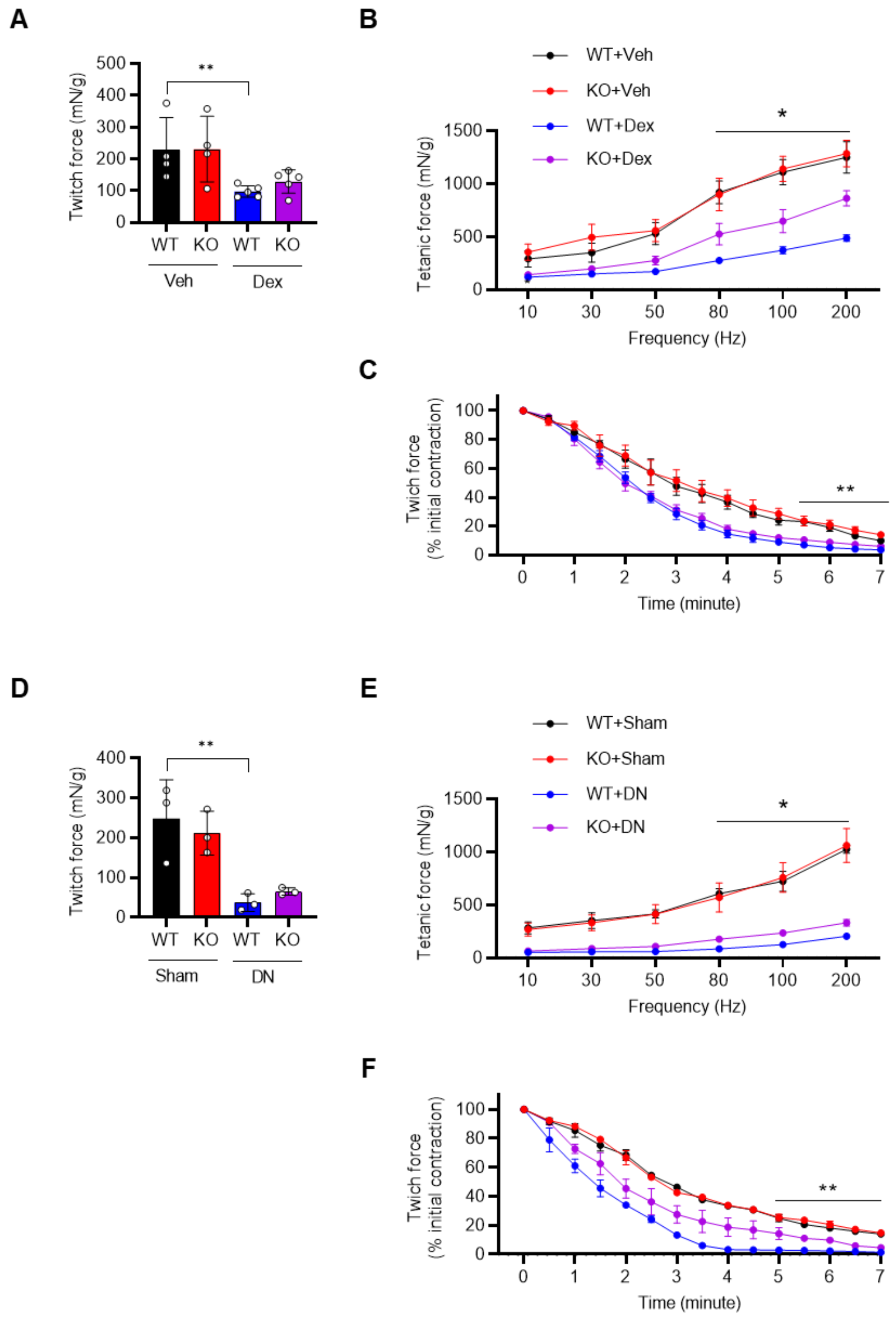

Figure S9. Enhanced force production and decreased muscle fatigue in gastrocnemius muscles from *Pak4* KO mice. Gastrocnemius muscles were isolated from dexamethasone (Dex)- (A-C, n = 4 for Veh, n = 5 for Dex) and denervation (DN)-induced atrophy mice (D-F, n = 3 per each group). (A, D) After mounting the gastrocnemius muscles on a force transducer, twitch force was measured by electrically stimulating the muscle with a single pulse (100 V for 1 ms). (B, E) The tetanic force–frequency relationships were determined by inducing contractions with incremental stimulation frequencies (1 ms pulses at 10–200 Hz for 500 ms at 100 V). (C, F) Fatigue index was measured at 1 Hz and 100 V using repeated stimuli for 7 minutes and expressed as a percentage of the initial contractile force. Values are mean ± SD. *, $p<0.05$ and **, $p<0.01$.

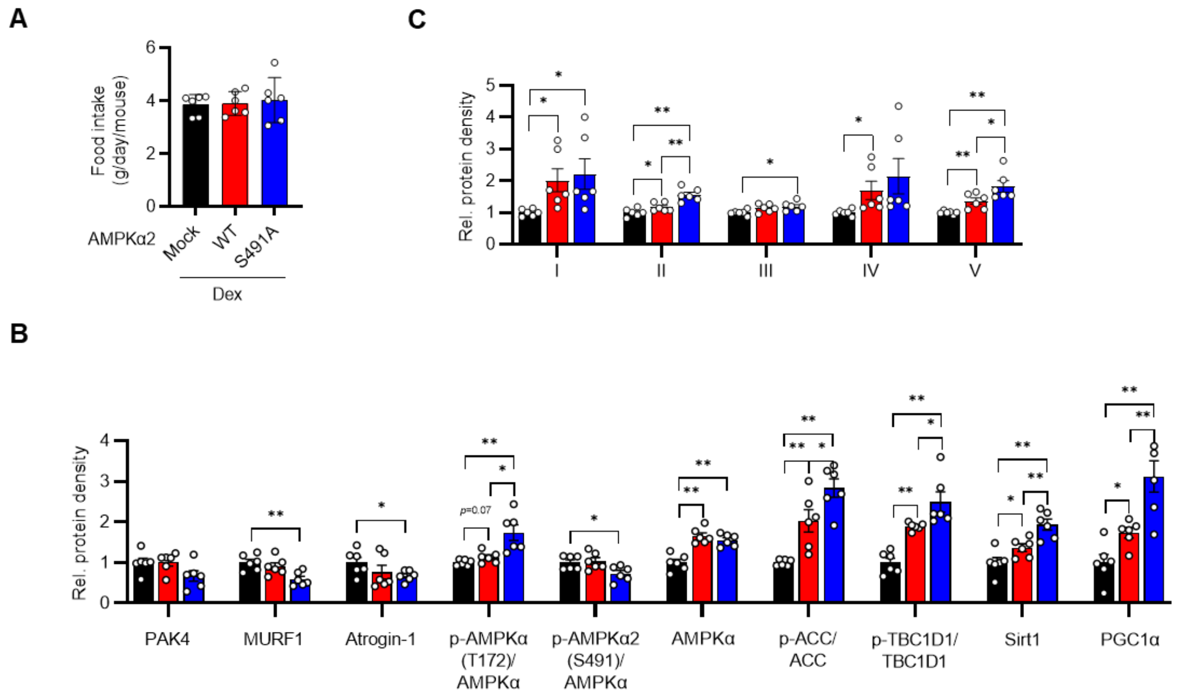

Figure S10. Attenuation of muscle atrophy in skeletal muscle-specific overexpression of AMPKα2^{S491A}. Experimental procedures were identical to those described in the Figure 5 legend. (A) Daily food intake. (B, C) Quantification of Western blot band intensities corresponding to Fig. 3E (B) and 3F (C). Values are mean ± SD (*n* = 6 per each group). *, $p<0.05$ and **, $p<0.01$.

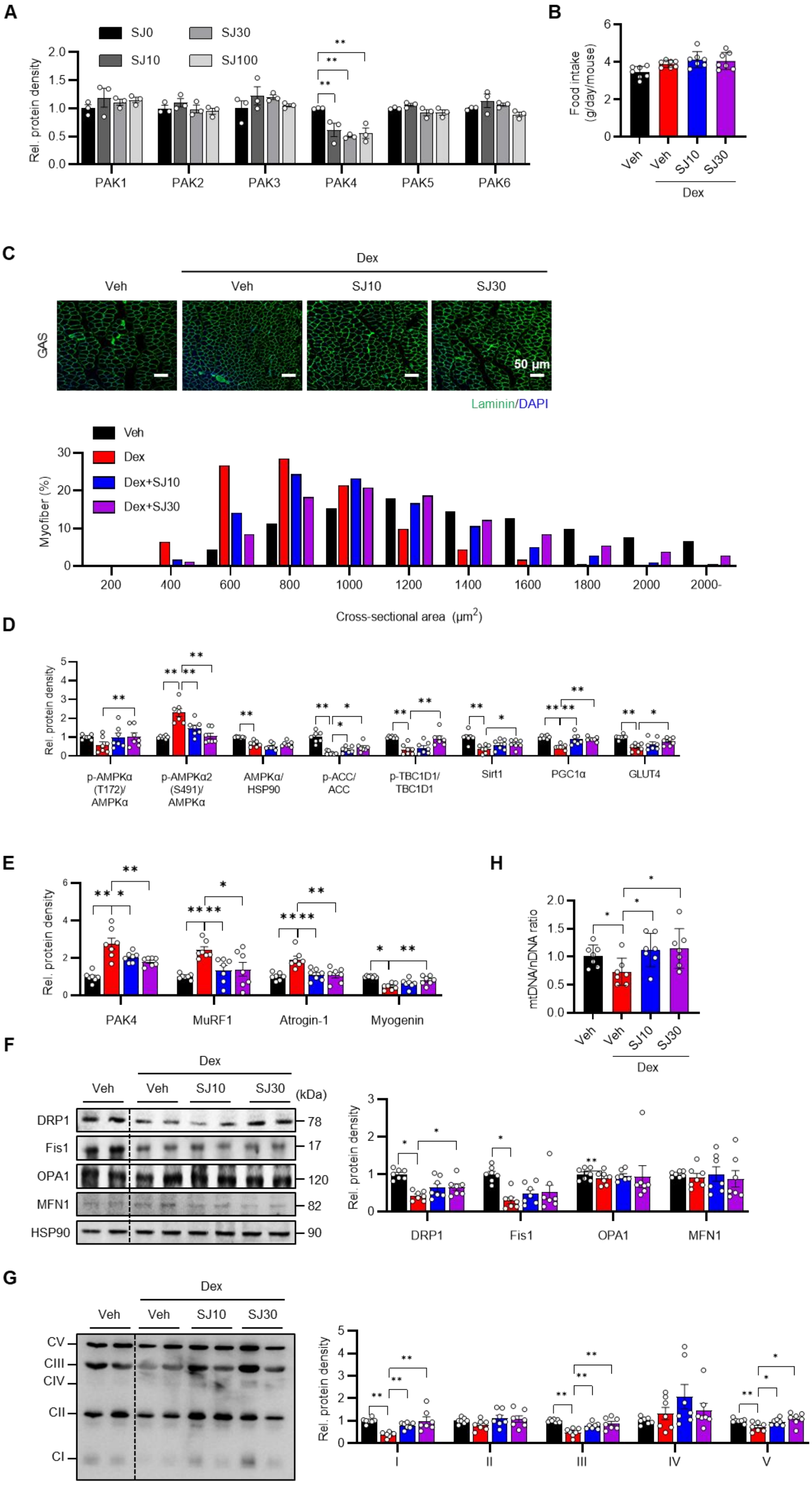

A
SJ0
SJ30
SJ10
SJ100
Rel. protein density
PAK1
PAK2
PAK3
PAK4
PAK5
PAK6
B
Food intake (g/day/mouse)
Veh
Veh
SJ10
SJ30
Dex
C
Dex
Veh
Veh
SJ10
SJ30
GAS
50 μm
Laminin/DAPI
Veh
Dex
Dex+SJ10
Dex+SJ30
Myofiber (%)
Cross-sectional area (μm2)
D
Rel. protein density
p-AMPKα (T172)/ AMPKα
p-AMPKα2 (S491)/ AMPKα
AMPKα/ HSP90
p-ACC/ ACC
p-TBC1D1/ TBC1D1
Sirt1
PGC1α
GLUT4
E
Rel. protein density
PAK4
MuRF1
Atrogin-1
Myogenin
H
mtDNA/nDNA ratio
Veh
Veh
SJ10
SJ30
Dex
F
Dex
Veh
Veh
SJ10
SJ30
(kDa)
DRP1
Fis1
OPA1
MFN1
HSP90
Rel. protein density
DRP1
Fis1
OPA1
MFN1
G
Dex
Veh
Veh
SJ10
SJ30
CV
CIII
CIV
CII
CI
Rel. protein density
I
II
III
IV
V

Figure S11. Mitigation of dexamethasone-induced muscle atrophy by PAK4 PROTAC. Experimental procedures were identical to those described in the Figure 6 legend. (A) Quantification of Western blot band intensities corresponding to Fig. 6A (n = 3). SJ0, vehicle; SJ10, SJ 10 nM; SJ30, SJ 30 nM; SJ100, SJ, 100 nM. (B) Daily food intake (n = 7 per each group). (C) Immunofluorescence staining of laminin in muscle sections. Myofiber cross-sectional area was quantified (n = 7 per each group). Bar=50 µm. (D, E) Quantification of Western blot band intensities corresponding to Fig. 6F (D) and 6G (E) (n = 7 per each group). (F, G) Western blot analysis of mitochondrial dynamics (F) and OxPhos (G) proteins. (H) Mitochondrial DNA (mtDNA) was quantified by qPCR using nuclear DNA (nDNA) as a reference (n = 7 per each group). Values are mean ± SD. *, $p<0.05$ and **, $p<0.01$.

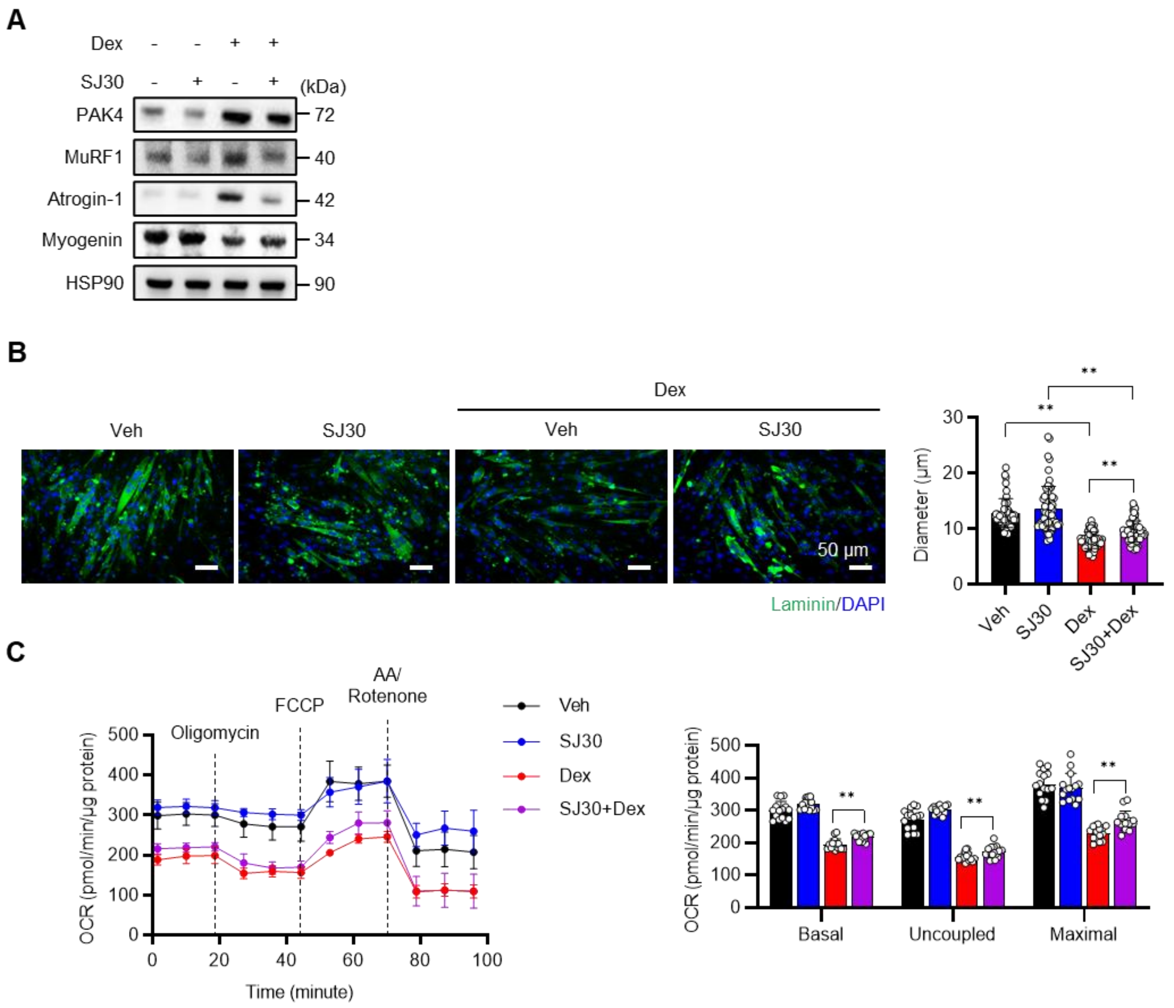

Figure S12. PAK4 PROTAC alleviates dexamethasone-induced atrophic changes in C2C12 cells. C2C12 cells were treated with 1 μM dexamethasone, with or without 30 nM SJ-05 (SJ30) for 24 hours. Atrogenes were analyzed by Western blotting (*n* = 3). (B) C2C12 cells were immunostained with anti-laminin antibody, and myofiber diameter was quantified (*n* = 58-62). Bar = 50 μm. (C) The oxygen consumption rate (OCR) in C2C12 cells was measured using a Seahorse XF analyzer. Basal respiration, ATP production-related respiration (uncoupled, calculated as the difference between OCR before and after oligomycin injection), and maximal respiration (difference between OCR after FCCP and after antimycin A (AA)/rotenone injection) were determined (*n* = 15). Values are mean ± SD. **, p<0.01.

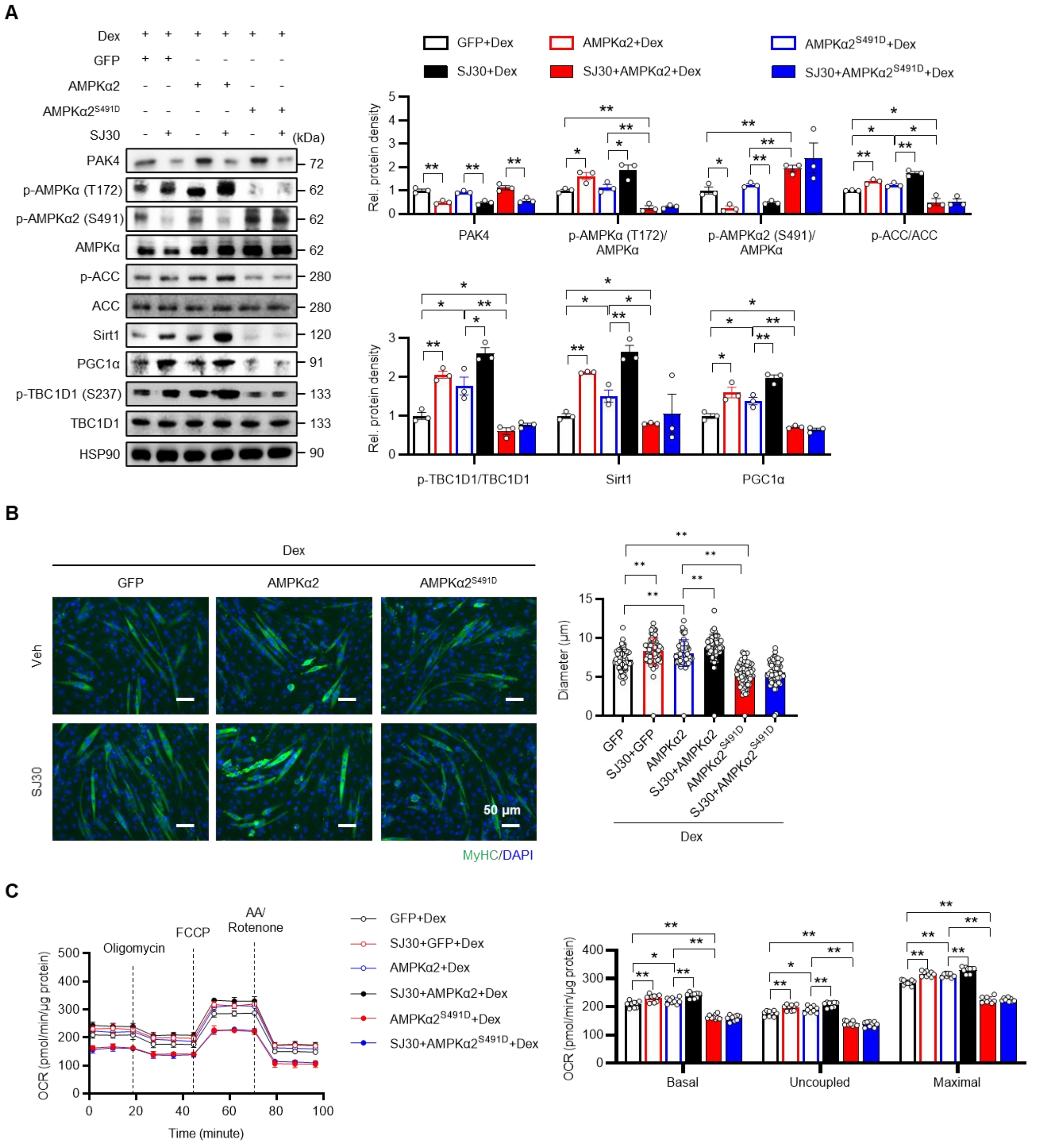

Figure S13. Reduction of JS-05 effects in C2C12 cells by AMPKα^{S491D} overexpression. C2C12 cells were transfected with either AMPKα2 or MPKα2^{S491D} and treated with 1 μM dexamethasone, with or without 30 nM SJ-05 (SJ30), for 24 hours. (A) Activation of AMPKα-related pathway was analyzed by Western blotting (n = 3). (B) Cells were immunostained with anti-MyHC antibody, and myofiber diameter was quantified (n = 60-68). Bar = 50 μm. (C) The oxygen consumption rate (OCR) in C2C12 cells was measured using a Seahorse XF analyzer (n = 9). Values are mean ± SD. *, p<0.05 and **, p<0.01.

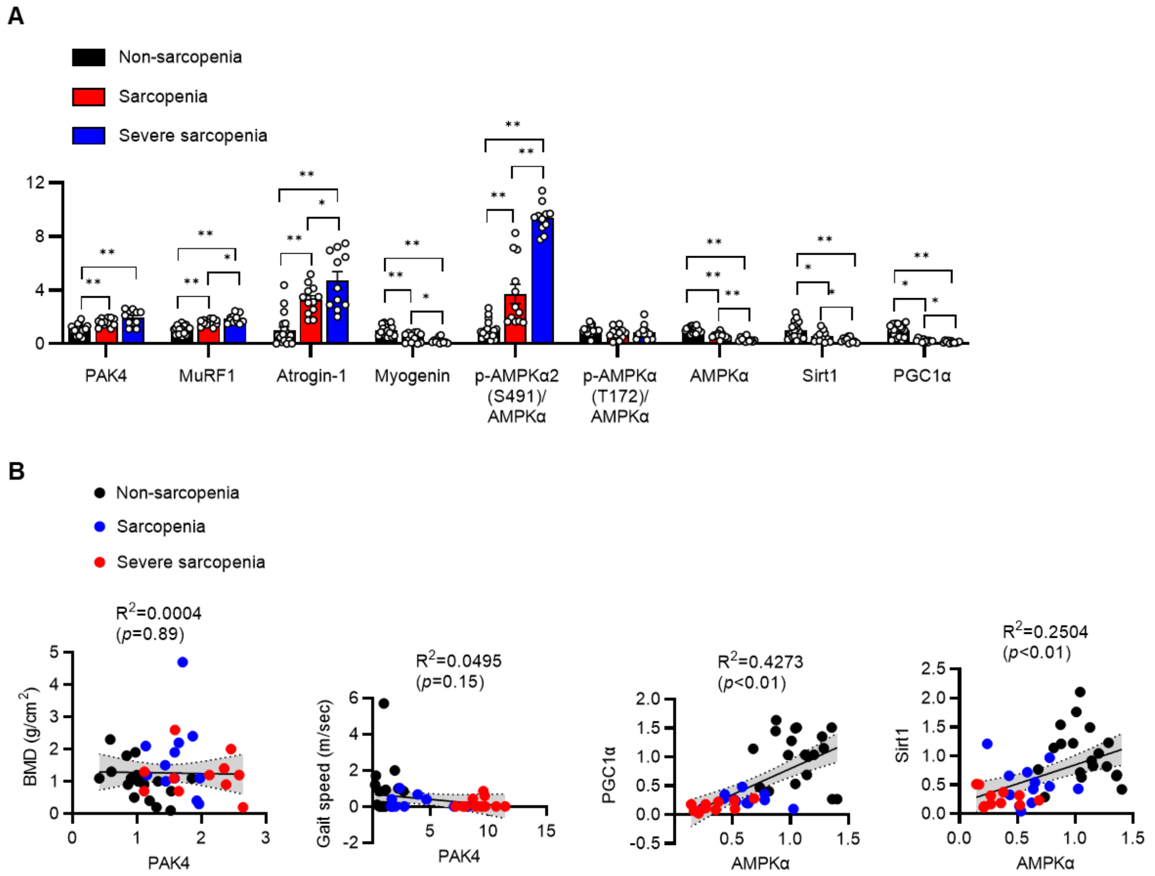

Figure S14. Relationship between key protein expressions in skeletal muscle tissues and sarcopenic parameters in humans. Experimental procedures were identical to those described in the Figure 7 legend. (A) Quantification of Western blot band intensities corresponding to Fig. 7A (*n* = 19 for non-sarcopenia, *n* = 12 for sarcopenia, *n* = 11 for severe sarcopenia). (B) Correlation between the PAK4 or AMPKα and the sarcopenic parameters, PGC1α, or Sirt1 (*n* = 42). Values are mean±SD. *, p<0.05 and **, p<0.01. BMD, bone mineral density.

3. Supplementary Tables

Table S1. Clinicopathologic variables in muscle donors

Characteristics	Non-sarcopenia		Sarcopenia		*p* value*	Severe sarcopenia		*p* value#	*p* value&
	Mean	SD	Mean	SD		Mean	SD		
Sex (M/F)	12/7	-	5/7	-		2/9			
Age (year)	62.37	13.09	65.83	19.84	0.7821	78.64	5.07	0.0108	0.0860
RSMI (kg/m^2)	8.17	2.50	5.44	1.06	0.0007	4.97	0.79	0.0001	0.8092
BMD (g/cm^2)	1.04	0.54	1.66	1.12	0.1039	1.20	0.63	0.8457	0.3770
Gait speed (m/sec)	0.77	1.32	0.22	0.33	0.2720	0.18	0.29	0.2467	0.9953
Grip strength (kg)	26.86	8.59	14.18	10.47	0.0018	10.86	7.45	0.0001	0.6678

* indicates a comparison between the non-sarcopenia and sarcopenia groups; # denotes a comparison between the non-sarcopenia and severe sarcopenia groups; & signifies a comparison between the sarcopenia and severe sarcopenia groups. RSMI, relative skeletal muscle index; BMD, bone mineral density.

Table S2. Antibodies used for western blotting, immunofluorescence and immunohistochemical analyses

Antibody	Catalogue No.	Dilution	Company
Western blot			
PAK4 (G222)	62690	1:2500	Cell Signaling Technology
HSP90 (C45G5)	4877	1:2500	Cell Signaling Technology
PAK1	2602	1:2500	Cell Signaling Technology
PAK2	2608	1:2500	Cell Signaling Technology
PAK3	2609	1:2500	Cell Signaling Technology
PAK5	62234	1:2500	Cell Signaling Technology
p-AMPKα (Thr172) (40H9)	2535	1:2500	Cell Signaling Technology
AMPKα (D5A2)	5831	1:2500	Cell Signaling Technology
AMPKα2	2757	1:2500	Cell Signaling Technology
p-Akt (Ser473) (D9E)	4060	1:2500	Cell Signaling Technology
Akt2 (D6G4)	3063	1:2500	Cell Signaling Technology
Sirt1	2310	1:2500	Cell Signaling Technology
PGC1α	AB3242	1:2500	Merck
p-PKA Substrate (RRXS*/T*) (100G7E)	9624	1:2500	Cell Signaling Technology
p-p70 S6 Kinase (Thr389)	9205	1:2500	Cell Signaling Technology
p70 S6 Kinase	9202	1:2500	Cell Signaling Technology
PAK6 Antibody (H-300)	sc-32857	1:2500	Santa Cruz Biotechnology
ACCα (H74)	BS1378	1:2500	Bioworld Technology
Anti-AMPKα2 (p-S491)	ab109402	1:2500	Abcam
Atrogin-1	ab168372	1:2500	Abcam
MuRF1	sc-398608	1:2500	Santa Cruz Biotechnology
Myogenin	sc-12732	1:2500	Santa Cruz Biotechnology
GLUT4	sc-53566	1:2500	Santa Cruz Biotechnology
DRP1	sc-271583	1:2500	Santa Cruz Biotechnology
Fis1	sc-376447	1:2500	Santa Cruz Biotechnology
OPA1	sc-393296	1:2500	Santa Cruz Biotechnology
MFN-1	sc-166644	1:2500	Santa Cruz Biotechnology
OXPHOS	ab110413	1:2500	Abcam
PTEN	sc-7974	1:2500	Santa Cruz Biotechnology
p-PI3K	4228	1:2500	Cell Signaling Technology
PI3K	4292	1:2500	Cell Signaling Technology
p-FoxO3a	9466	1:2500	Cell Signaling Technology
FoxO3a	12829	1:2500	Cell Signaling Technology
p-mTOR	2971	1:2500	Cell Signaling Technology
mTOR	2972	1:2500	Cell Signaling Technology
p-TBC1D1 (Ser237)	# PA5-99563	1:2500	Invitrogen
TBC1D1	#22124-1-AP	1:2500	Proteintech
Immunofluorescence			
MF-20	11001	1:100	DSHB
Laminin	L9393	1:100	Merck
Alexa Fluor 594-conjugated goat anti-rabbit IgM	11012	1:100	Thermo Fisher Scientific

Table S3. Information for primers (forward, FOR; reverse, REV)

Gene (qPCR)	Sequences for primers	Accession No.
Pak4	FOR: GCTCCCCTTTGAAGATGTCA<br>REV: GACCCACAAGGACTCAAGGA	NM_027470
Trim63	FOR: CCAGGCTGCGAATCCCTAC<br>REV: ATTTTCTCGTCTTCGTGTTCCTT	NM_010493
Fbxo32	FOR: CAGCTTCGTGAGCGACCTC<br>REV: GGCAGTCGAGAAGTCCAGTC	NM_013528
